# Structure and mechanism of HDV ribozymes revealing Lewis acid catalysis by a metal ion

**DOI:** 10.1038/s41467-026-77452-w

**Published:** 2026-09-02

**Authors:** Yuhang Luo, Xiaoxue Chen, Xiaowei Lin, Wenjian Liao, Bowen Xiao, Mengxiao Li, Zhaoji Qiu, Timothy J. Wilson, Zhichao Miao, Jia Wang, Lin Huang, David M. J. Lilley

**Affiliations:** 1 Guangdong Provincial Key Laboratory of Malignant Tumor Epigenetics and Gene Regulation, Guangdong-Hong Kong Joint Laboratory for RNA Medicine, Sun Yat-Sen Memorial Hospital, Sun Yat-Sen University, Guangzhou, China; 2 Department of Pharmacy, Sun-Yat-Sen Memorial Hospital, Sun Yat-sen University, Guangzhou, China; 3 Department of Urology, Dafeng Hospital, Shantou, China; 4 Department of Urology, The Third Affiliated Hospital of Southern Medical University, Guangzhou, China; 5 Guangzhou National Laboratory, Guangzhou, China; 6 Zhongshan School of Medicine, Sun Yat-sen University, Guangzhou, China; 7 School of Life Sciences and Biopharmaceutics, Guangdong Pharmaceutical University, Guangzhou, China; 8 Nucleic Acid Structure Research Group, The Division of Molecular, Cell and Developmental Biology, Faculty of Life Sciences, The University of Dundee, Dundee, UK; 9 College of Pharmacy, Shenzhen Technology University, Shenzhen, China

**Keywords:** Biocatalysis, X-ray crystallography

## Abstract

We have determined the molecular structure and investigated the catalytic mechanism of two ribozymes of the Hepatitis delta virus family, found in the nematode *Caenorhabditis briggsae* and virus *Ackermannviridae*. Crystal structures of both conform to the double-pseudoknot architecture adopted by the viral HDV ribozyme. The *C. briggsae* ribozyme has been determined both pre- and post-cleavage. In the former both nucleotides flanking the scissile phosphate are observed, along with a metal ion, and cytosine 75 N3 bound to the O5’ leaving group. The pH dependence of cleavage rate reveals a p*K*_a_ of 6.5 and together with the inactivity of a C75U mutant provides evidence for its role as general acid. In contrast to other nucleolytic ribozymes that use catalytic metal ions, reaction rate does not depend on the p*K*_a_ of the divalent metal ion. Limited adjustment of structure of the active center is consistent with direct bonding of the metal ion to the O2’ and non-bridging O, suggesting that the ion acts as a Lewis acid to activate nucleophilic attack. This mechanism appears to be general for the HDV ribozyme class, and distinguishes it from the majority of nucleolytic ribozymes that use general base catalysis.

## Abstract (web summary)

The HDV ribozyme catalyzes rapid cleavage of RNA. Here the authors provide crystallographic and mechanistic analysis showing that the ribozyme accelerates the reaction in part using Lewis acid catalysis by a bound magnesium ion in the active center. This contrasts with other known nucleolytic ribozymes.

## Introduction

RNA catalysis is important from several perspectives. Some key reactions in the cell are catalyzed by RNA including mRNA splicing^1^ and peptidyl transfer^2,3^. Some ribozymes are widely encoded in genomes^4–7^. According to the RNA World hypothesis^8,9^, RNA catalysis would have played a key role in the origin of life on the planet. Yet the chemical origins of RNA catalysis are incompletely understood.

In broad terms, ribozymes catalyzing phosphoryl transfer reactions can be divided into two major mechanistic classes. The larger ribozymes exemplified by the group I intron ribozyme^10–15^ are metalloenzymes where two or three bound metal ions organize the active center and activate catalysis. This class does not use general acid-base catalysis. The second group contains the nucleolytic ribozymes, a group of around 12 ribozymes that undergo site-specific cleavage by a transesterification reaction initiated by attack of a particular 2’-hydroxyl group on the adjacent phosphodiester linkage^16,17^. In contrast to the intron ribozymes, most nucleolytic ribozymes use general base-acid catalysis, employing nucleobases, the 2’-hydroxyl group or hydrated metal ions. However, questions remain on how general this might be, and particularly with regard to the importance of general base catalysis^18^.

Hepatitis delta virus is a human pathogenic virus that causes severe hepatitis. It is a satellite RNA of the hepatitis B virus that is required to enable viral replication in cells. HDV replicates by a rolling circle mechanism, generating single-stranded genomic and antigenomic circular RNA species. Each includes the sequence of a ~ 85 nt closely-similar nucleolytic ribozyme, the activities of which generate monomeric linear RNA species^19,20^. The ribozymes were found to adopt secondary structures comprising five helical sections connected in a nested double pseudoknot structure. In common with other nucleolytic ribozymes, they undergo site-specific cleavage generated by attack of a specific 2’-hydroxyl group on the adjacent phosphorus atom to generate cyclic 2’3’-phosphate and 5’-hydroxyl groups flanking the cleavage site.

**Fig. 1:**
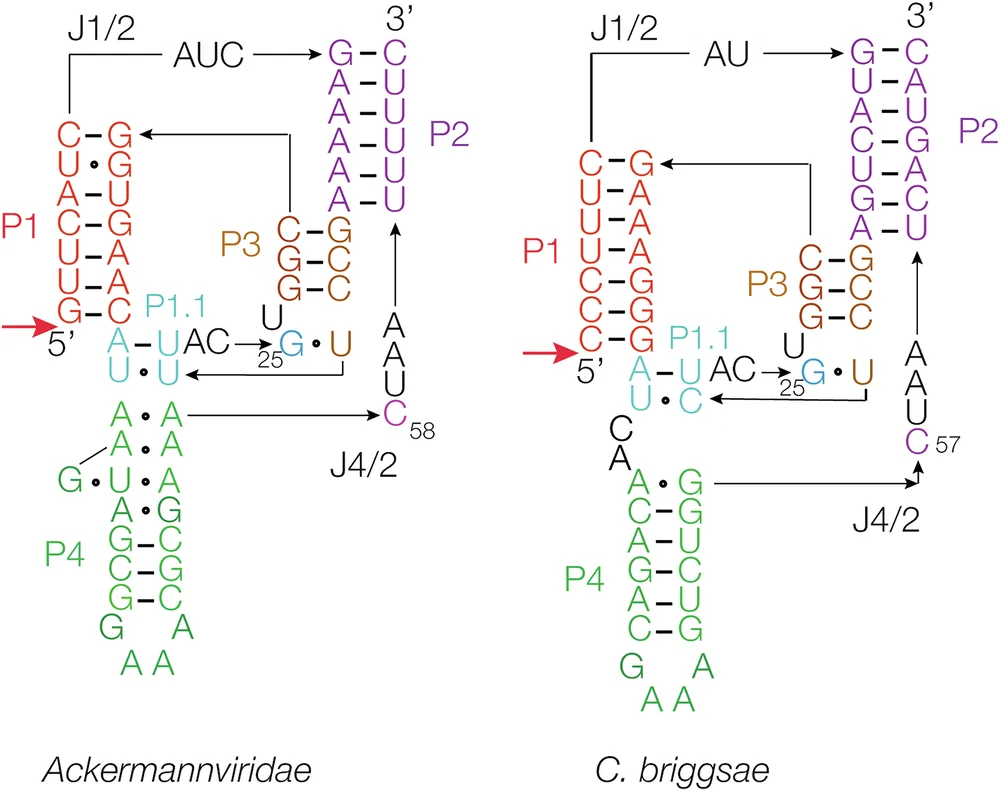
Comparison of ribozyme sequence and secondary structure for the HDV-like ribozyme sequences from *Ackermannviridae* and *C.* ***briggsae***. The red arrows denote the predicted site of ribozyme cleavage. The expected secondary structure is colored consistently with the structural data reported here.

It was subsequently found that HDV ribozyme-like sequences occurred more widely. Szostak and co-workers^21^ employed an in vitro selection method to isolate several self-cleaving RNA species from the human genome, one of which was clearly closely related to the HDV ribozyme. This was found in an intron of the *CPEB3* gene and was conserved in all mammals. Using the double-pseudoknot architecture as the basis for structure-based searching subsequently led to the discovery of many more HDV-like putative ribozymes, from lamprey, lancelet, nematode, sea urchin, insects and even bacteria^22,23^. Some were found to be active in vivo, but in general, rates of cleavage were observed to be slow. For example, the human CPEB3 ribozyme was reported to cleave with a rate constant of *k*_obs_ = 0.012 min^−1^. However, cleavage could be accelerated by modification of peripheral sequences^24^, indicating that slow cleavage was not intrinsic to the CPEB3 ribozyme structure. More recently, Breaker and colleagues^25^ used a computational pipeline involving the sequential use of BLAST sequence search followed by an analysis of secondary structure. In this work we focused on two representatives emerging from that analysis for this study (Fig. 1), and verified their origins via NCBI BLAST. The first sequence is derived from a dsDNA virus belonging to the *Ackermannviridae* family, a bacteriophage known to infect *Enterobacteriaceae*. The second is from the eukaryotic nematode *Caenorhabditis briggsae*.

The structure of the viral HDV ribozyme has been determined both before and after cleavage by X-ray crystallography in a number of investigations^26–30^. These confirmed the double-pseudoknot architecture of the RNA, with two coaxial stacks of helices (P1-P1.1-P4 and P2-P3) packed side by side. The catalytic mechanism of the viral ribozyme has also been studied in detail. The pH dependence of the cleavage rate indicated that a group with a p*K*_a_ = 6.6 was catalytically important^31^, assigned to a key cytosine (C75 in the viral genomic enzyme sequence). While a C75U mutant was inactive in cleavage, activity could be restored by exogenous imidazole^32^, and the C75 nucleobase is located adjacent to the O5’ leaving group in the crystal^29^. This suggested a role for C75 as a general acid to protonate the 5’ oxyanion leaving group, and this was established definitively by Das and Piccirilli using phosphorothiolate substitution^33^. The other potential participant in the catalytic chemistry is a divalent metal ion. Ions have been observed in the catalytic center of the viral ribozyme^27,28^, and a stereospecific phosphorothioate effect was observed at the *pro*-R non-bridging oxygen atom of the scissile phosphate^34^. In principle, a metal ion might activate nucleophilic attack by binding as a Lewis acid, or an inner-sphere water molecule might act as a general base ^29,31,35,36^. This issue has not been fully resolved.

For the majority of the non-viral HDV-like ribozymes, only sequence information is available. For most, there is no structural information (with the exception of some primate CPEB3 ribozymes^37^), and there has been almost no mechanistic investigation of any of these ribozymes. It is important to explore the generality of the structural and mechanistic principles established for the viral HDV ribozyme, and we have therefore solved crystal structures for the HDV ribozymes of *C. briggsae* and *Ackermannviridae* identified by Weinberg et al.^25^, both before and after the cleavage reaction in one case. We have performed a detailed mechanistic investigation of these ribozymes. Our data support the role of cytosine nucleobase-mediated general acid catalysis. We observe bound Mg^2+^ ions, and the absence of a dependence of reaction rate on metal ion p*K*_a_ (in contrast to that observed for other metal ion-utilizing nucleolytic ribozymes) indicates that the major role is activation of nucleophilic attack acting as a Lewis acid.

## Results

### Two new HDV ribozyme sequences

In this study we have investigated the three-dimensional molecular structure and catalytic mechanism of the *C. briggsae* (strain AF16, chromosome V, GenBank Accession No. CP120834.1) and *Ackermannviridae* (isolate ctUKy2, GenBank Accession No. BK037802.1) HDV-like ribozymes (Fig. 1). These two ribozyme sequences were selected on the basis of sequence and structural diversity of the newly identified HDV ribozyme variants reported by Breaker and colleagues^25^, with particular emphasis on differences in the P4 stem architecture. Specifically, the candidate set included ribozymes with distinct P4 stem features, including variants containing an E-loop motif and those lacking it. Thus, these RNAs were chosen to sample naturally occurring structural diversity within the HDV-like ribozyme family. In addition, these two sequences yielded crystals that diffracted to sufficiently high resolution for detailed structural analysis.

### Division of the new HDV ribozymes into ribozyme plus substrate to analyze cleavage kinetics

**Fig. 2:**
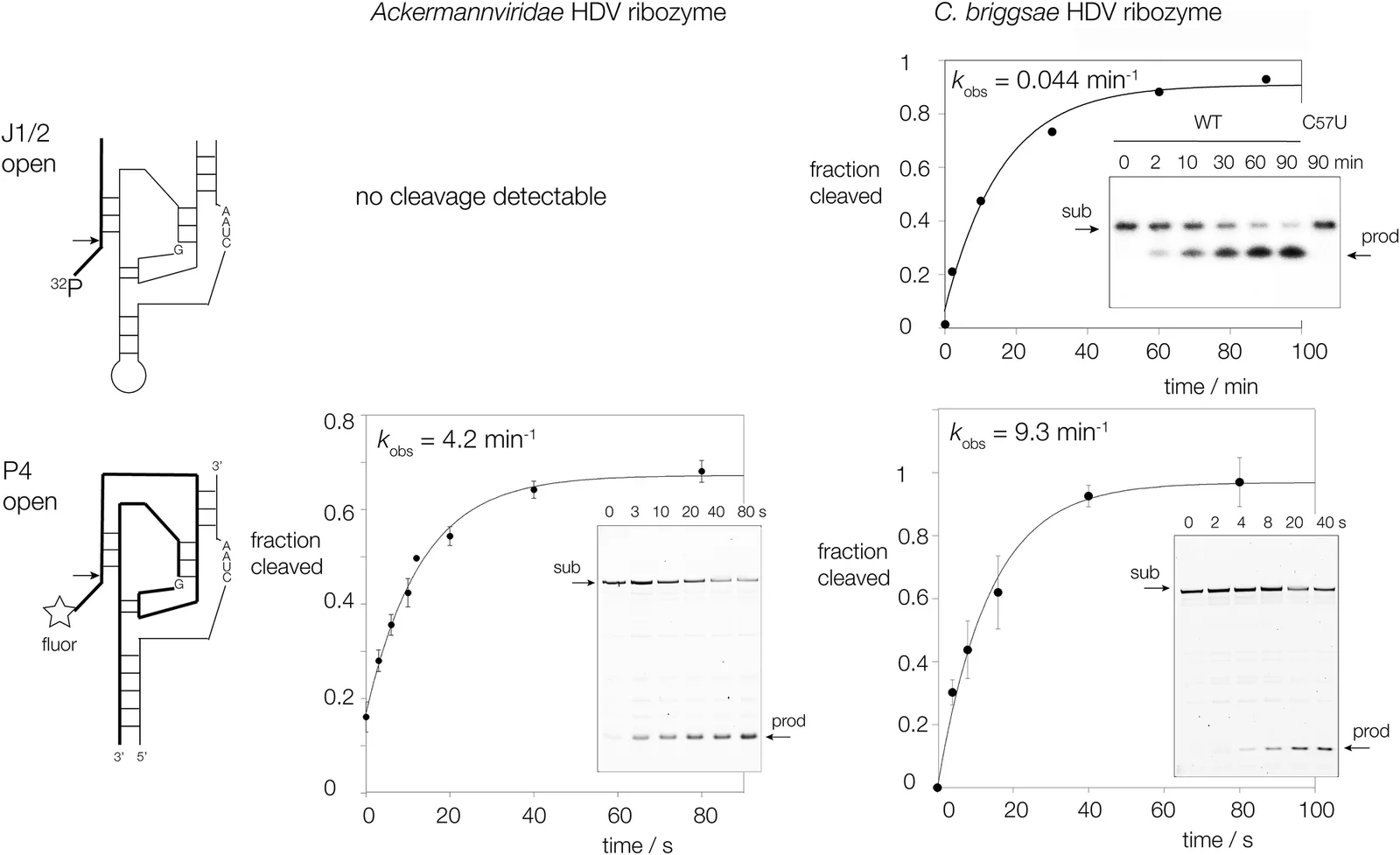
Cleavage of *Ackermannviridae* and *C*. *briggsae* HDV ribozymes as a function of the manner of dividing into two RNA species. In the upper constructs the RNA has been divided at the J1/2 connection between P1 and P2. In the lower constructs, the P4 helix has been extended and its terminal loop opened. In these schematics, the base pairing bars do not represent the exact number of base pairs. For each construct that exhibits cleavage activity, the fraction of cleaved substrate RNA is plotted as a function of time, and the data are fitted to a single exponential function. The points are mean values, and the error bars are standard deviations. The calculated rate constants and the number of replicates are shown in Table 1. The gel electrophoresis separating substrate (sub) and product (prod) is shown as an insert, with times of incubation indicated.

In the mechanistic dissection of nucleolytic ribozyme action it is experimentally advantageous to divide the RNA into two sections that can be considered to be the ribozyme and substrate, and perform cleavage reactions under single-turnover conditions. Our standard reaction conditions are 20 mM Tris (pH 7.5), 2 mM Mg^2+^ ions, using 1 µM ribozyme and 150 nM substrate strands. Past experience shows that care must be exercised in how ribozymes are engineered^38–41^, as this can readily result in a considerable loss of activity such that the catalytic chemistry may not be rate-limiting. We divided *Ackermannviridae* and *C. briggsae* ribozymes according to two alternative strategies (Fig. 2). These were:

1. *Breaking the connection between the P1 and P2 helices (J1/2)*. This strategy was used by Das and Piccirilli^33^ for the analysis of the original human viral HDV ribozyme, who reported a rate of 1 min^−1^ at pH 7.5. We studied the corresponding constructs (Supplementary Table S1), generating the longer (ribozyme) strands using transcription from a PCR-generated template, and the shorter (substrate) strands by chemical synthesis. The substrate strands were radioactively [5’-^32^P]-labeled. No activity was detected for the *Ackermannviridae* HDV ribozyme. The *C. briggsae* ribozyme exhibited cleavage activity, but with an observed rate corresponding to *k*_obs_ = 0.044 ± 0.002 min^−1^ (Fig. 2).

2. *Breaking the covalent continuity of helix P4*. Given the poor activity of the above forms of the new HDV ribozymes, we adopted a second strategy in which the P4 helix was extended by 3 bp and the terminal loop opened (Fig. 2; Supplementary Table S1). The 5’ end was extended by seven nucleotides from the expected cleavage site, so that the total length of the 5’ RNA was 56 nt, and that of the 3’ RNA was 20 nt. Both RNA species were generated by chemical synthesis. We attached a fluorophore to the 5’ terminus for detection. The labeled 56 nt strand becomes shortened to 7 nt by ribozyme cleavage. When divided in this manner, the *Ackermannviridae* HDV ribozyme was now active, with an observed rate corresponding to *k*_obs_ = 4.2 ± 0.44 min^−1^ (Table 1). The *C. briggsae* HDV ribozyme exhibited strong cleavage activity, cleaving at a unique site with an observed rate of characterized by *k*_obs_ = 9.3 ± 1.0 min^−1^. Both *Ackermannviridae* and *C. briggsae* HDV ribozymes cleave at the expected position for ribozyme action. We chemically synthesized the predicted 7 nt fragments and observed that their migration in polyacrylamide under denaturing conditions is similar to those of the products of ribozyme cleavage (Supplementary Fig. S1). The products of ribozyme cleavage migrate fractionally faster than the synthetic RNA because they terminate with a cyclic 2’3’ phosphate, thus adding an additional charge.

**Table 1:** Rate constants for cleavage by HDV ribozymes under single-turnover conditions

ribozyme	conditions*	rate /min^−1^	SD	number of replicates
***Ackermannviridae*** **ribozyme**				
J1/2 open		N/A		
P4 open		4.6	0.40	5
P4 open C58U		N/A		
***C. briggsae*** **ribozyme**				
J1/2 open WT		0.044	0.0016	5
P4 open WT		9.3	1.01	4
P4 open WT	2 mM Ca^2+^	11.1	1.45	4
P4 open WT	2 mM Mn^2+^	6.7	1.59	4
P4 open WT	MES (pH 6.0)	2.4	0.29	4
P4 open WT	MES (pH 6.7)	5.1	0.50	3
P4 open WT	Tris (pH 8.5)	9.9	1.12	4
P4 open C57U		N/A		
P4 open G25O^6^S		0.010	0.0016	3

* other than standard conditions 20 mM Tris.HCl (pH 7.5), 2 mM MgCl_2_.

*N/A* no activity detectable; *SD* standard deviation.

The rate of cleavage for the *C. briggsae* HDV ribozyme is fast for any nucleolytic ribozyme, comparable to the pseudoknot-containing hammerhead and pistol ribozymes^41^. We note that as nucleolytic ribozymes are engineered to improve RNA folding they tend to cleave at rates corresponding to *k*_obs_ ~ 10 min^−1^. We have therefore concentrated our mechanistic investigation on the *C. briggsae* HDV ribozyme.

### Crystal structures of the new HDV ribozymes

**Fig. 3:**
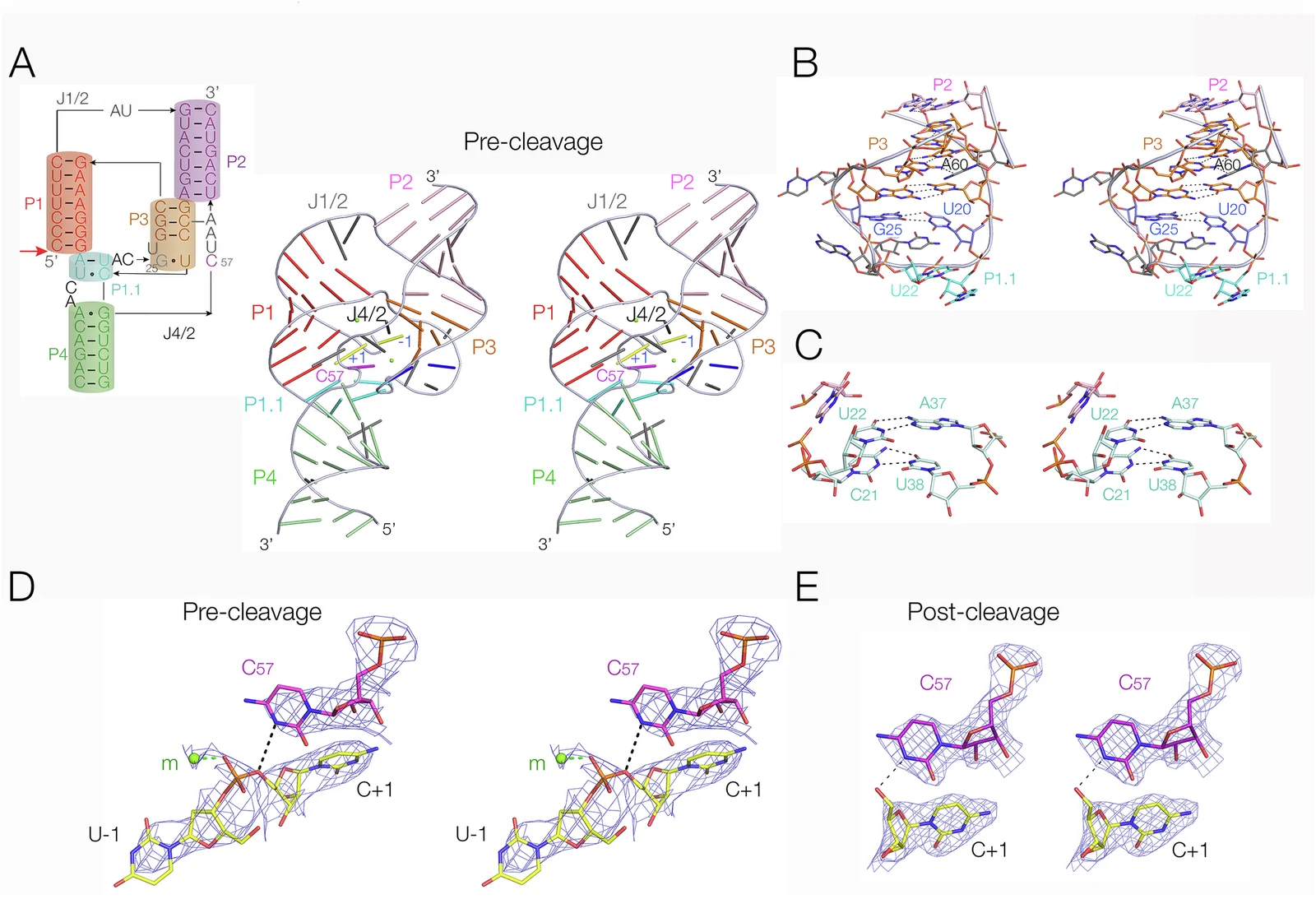
Crystal structure of the *C. briggsae* HDV ribozyme. **A** The overall structure of the ribozyme prior to cleavage. The scheme shown on the left shows the connectivity of the helices, and is colored to match the molecular structural images. The red arrow indicates the position of the cleavage reaction. On the right the structure is shown in cartoon form with bars representing the nucleotides for clarity. The molecular graphics are shown in parallel-eye stereoscopic representation. **B** The P2 P3 region of the structure. **C** The P1.1 pseudoknot helix. **D** The active site of the ribozyme prior to cleavage, with the 2**F**o-2**F**c electron density map contoured at 1.5σ. Note the presence of the Mg^2+^ ion (*m*) bonded to the *pro*-R non-bridging oxygen of the scissile phosphate, and C57 N3 in position to protonate the O5’ oxyanion leaving group. **E** The active site of the ribozyme after cleavage, with the 2**F**o-2**F**c electron density map contoured at 1.5σ. A bound Mg^2+^ ion is no longer observed, but the C57 N3 remains in position adjacent to the O5’ oxygen atom.

The *C. briggsae* HDV ribozyme was crystallized as both pre-cleavage and post-cleavage structures, in hexagonal and orthorhombic space groups, respectively (Supplementary Tables S2–4). The pre-cleavage ribozyme was generated by inclusion of a 2’-deoxyribose substitution at the −1 position (so removing the potential nucleophile for the cleavage reaction), and the RNA was crystallized in the space group P6_5_22, and crystals diffracted to a resolution of 2.95 Å. The pre-cleavage ribozyme comprises five helical structures P1, P2 and P4 and two pseudoknot helices P3 and the two-base pair P1.1, all being standard A-form helices. P1, P1.1 and P4 are coaxial, as are P2 and P3, thus forming two side-by-side domains (Fig. 3A, Supplementary Fig. S2). These are connected by linking segments J1/2, J3/1.1, J1.1/3, J3/1 and J4/2. J4/2 includes the functionally important C57 (corresponding to C75 in the genomic viral HDV) and the strand is juxtaposed with the major groove of P3 (Fig. 3B), including a single triple-base interaction between A60 and the C18:G28 base pair, making a single hydrogen bond between G28 N2 and A60 N3. This is the only multiple-base interaction in the whole ribozyme structure. The [P1, P1.1, P4] and [P2, P3] stacked segments are also firmly connected by the formation of the P1.1 pseudoknot helix at the base of [P2, P3] (Fig. 3C).

In the viral HDV ribozyme, the P1.1 helix comprises two Watson-Crick G:C base pairs. The P1.1 helix of the *C. briggsae* HDV ribozyme comprises a standard Watson-Crick U:A base pair and a *cis* Watson-Crick C: U base pair connected by two hydrogen bonds between UN3 to CN3, and CN4 to UO4. Low rates of cleavage by some CPEB3 HDV-like ribozymes have been attributed in part to weakened stability of the P1.1 helix by inclusion of a non-Watson-Crick pairing at the second position^21,23,24^, but the *C. briggsae* HDV ribozyme is amongst the fastest nucleolytic ribozymes despite its C:U base pair. The overall RNA structure is closely similar to that of the viral HDV ribozyme, with an RMSD = 1.77 Å (Supplementary Fig. S3A).

The post-cleavage *C. briggsae* HDV ribozyme crystallized in the space group C1 2 1, and crystals diffracted to a resolution of 2.23 Å. All the structural features of the pre-cleavage ribozyme are retained in the structure after cleavage (Supplementary Fig. S4), and the two structures can be superimposed with an RMSD = 1.27 Å (Supplementary Fig. S3B).

We also crystallized the *Ackermannviridae* HDV ribozyme in its post-cleavage form. The RNA crystallized in the space group P2_1_2_1_2, and crystals diffracted to a resolution of 1.90 Å. The secondary and tertiary structure of the ribozyme (Supplementary Fig. S5) is closely similar to that of the *C. briggsae* ribozyme and retains all the same conformational elements. The two post-cleavage structures can be superimposed with an RMSD = 3.41 Å (Supplementary Fig. S3C). In the *Ackermannviridae* ribozyme the P1.1 helix comprises a Watson-Crick U:A base pair above a *cis* Watson-Crick U:U base pair connected by hydrogen bonds U21N3 to U38O2 and U38 N3 to U21O4 (Supplementary Fig. S6). Like the *C. briggsae* ribozyme, the non-Watson-Crick base pair does not prevent the ribozyme from cleaving at a fast rate. However, this is the most significant difference between the viral and cellular HDV ribozymes, so we feel that it is likely that a P1.1 helix of lowered stability is the reason for both *Ackermannviridae and C. briggsae* HDV ribozymes exhibiting a greater sensitivity to breakage of the region linking the P1 and P2 helices compared to the viral ribozyme.

### The conformations of the active centers of the new HDV ribozymes

The conformations of the active center of the *C. briggsae* HDV ribozyme pre- and post cleavage are shown in Fig. 3D, E, respectively. In the structure of the ribozyme that has undergone cleavage, the nucleotide at the −1 position (5’ to the cleavage site) is not present. But in the pre-cleavage active center, the positions of both U-1 and C1 and the connecting phosphate group are clearly defined by the electron density. This is unusual because in most structures of viral HDV ribozyme, the density for the −1 nucleotide is not visible, because nothing holds this nucleotide in place. However, in the *C. briggsae* HDV ribozyme structure the crystallographic symmetry closely pairs each ribozyme molecule, and the interaction includes mutual formation of a Watson-Crick-Hoogsteen base pair between U-1 of one molecule with A23 of the other (Supplementary Fig. S7). In addition, the U-1 nucleobases of each molecule are stacked together. This then fixes the position of nucleotide −1 in the pre-cleaved state, but is essentially an artifact of the crystal lattice. As we discuss below, this holds the U-1 and C1 in a conformation that would not be ideal for in-line nucleophilic attack.

In both pre- and post-cleavage structures, the N3 of C57 is directed towards the O5’ atom. This is similar to that observed in the viral HDV ribozyme structures^29^. The position of C57 is consistent with a role as a general acid to protonate the leaving group of the cleavage reaction. A very similar position of the corresponding C58 is observed in the active center of the *Ackermannviridae* HDV ribozyme in its post-cleavage state (Supplementary Fig. S5C).

In the active center of the pre-cleavage structure of the *C. briggsae* HDV ribozyme we observe electron density indicating the position of a bound metal ion 2.1 Å from the *pro*-R non-bridging oxygen atom of the scissile phosphate (Fig. 3D and Supplementary Fig. S8). This is consistent with a directly-bound magnesium ion. A bound metal ion is not observed in either of the product complexes.

### Cleavage rate as a function of pH

**Fig. 4:**
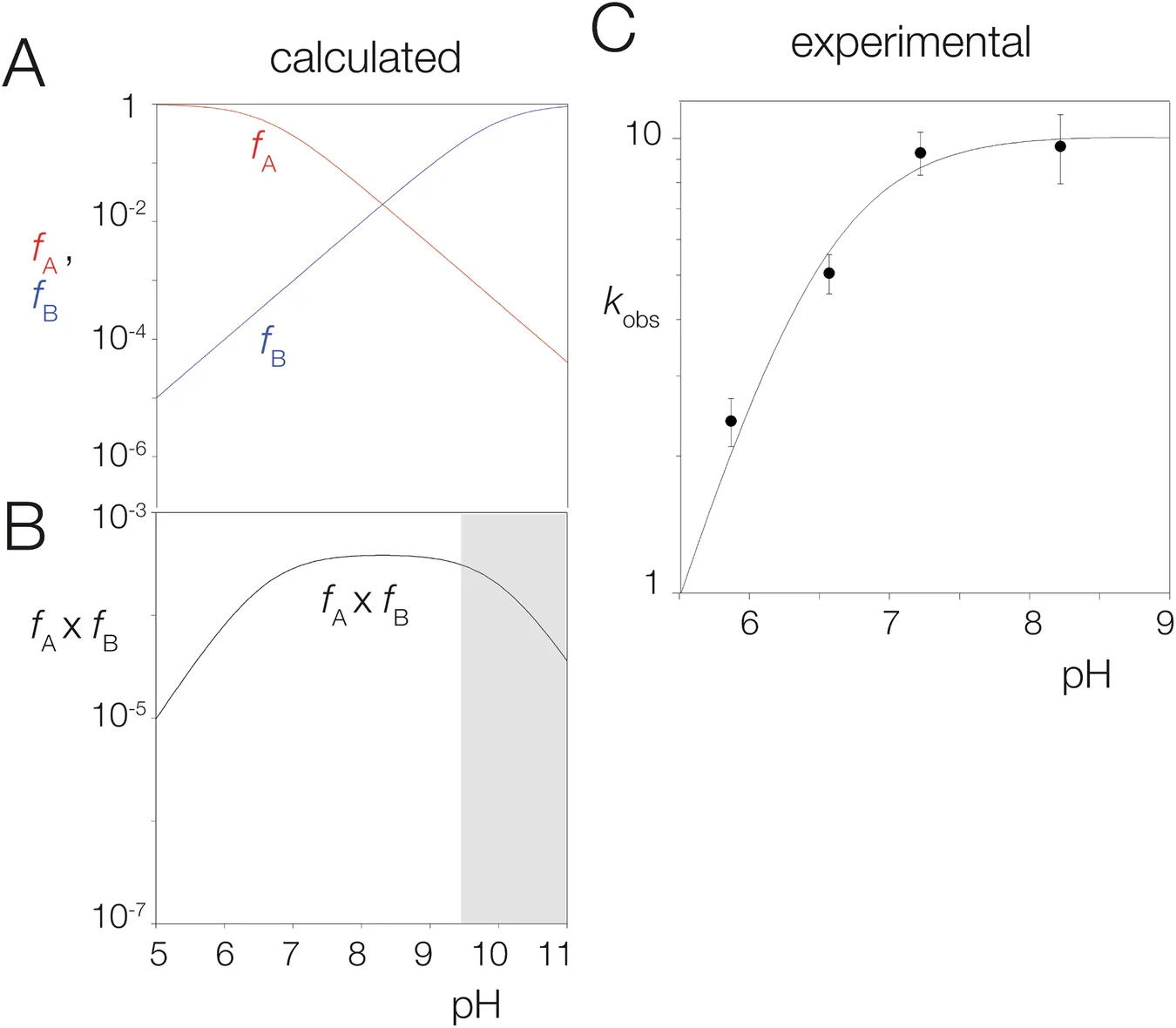
pH dependence of reaction rate. A simulation is shown on the *left*, and experimental data on the *right.* **A** The calculated fraction of protonated acid (*f*_A_ red, calculated for p*K*_a_ = 6.6) and deprotonated base (*f*_B_ blue, calculated for p*K*_a_ = 10) plotted as a function of pH. **B** The product *f*_A_ x *f*_B_ plotted as a function of pH. The section corresponding to pH > 9.5 is shown gray as this is not experimentally accessible due to the instability of the RNA at high pH. **C** The experimental pH dependence of the cleavage rate for the *C. briggsae* HDV ribozyme. The measured observed cleavage rate (*k*_obs_) is plotted as a function of pH, and fitted to a double ionization process. The rates have been measured multiple times, and the points are mean values, and the error bars indicate the standard deviations. The calculated rate constants and the number of replicates are shown in Table 1.

The anticipation that the new HDV ribozymes might use general acid-base catalysis leads to the prediction that the rate of cleavage will be dependent on solution pH. To be functional a general acid needs to be protonated, while a general base must be unprotonated. The observed rate of cleavage *k*_obs_ will therefore be given by

$${k}_{{{\rm{obs}}}}={k}_{{{\rm{cat}}}}.\,{f}_{{{\rm{A}}}}.{f}_{{{\rm{B}}}}$$

where *k*_cat_ is the intrinsic rate of cleavage (when the acid is protonated and the base unprotonated), *f*_A_ is the fraction of acid that is protonated, and *f*_B_ is the fraction of base that is unprotonated. The calculated values of *f*_A_ and *f*_B_ are plotted for the p*K*_a_ values of 6.6 (potentially a cytosine with a raised p*K*_a_ due to its environment) and 10 (i.e., a high p*K*_a_ that could be a hydrated metal ion for example), respectively (Fig. 4A). The profile of the cleavage reaction as a function of pH should follow that of the product *f*_A_. *f*_B_ as shown in Fig. 4B. We have measured the rate of cleavage by the *C. briggsae* ribozyme as a function of solution pH (Fig. 4C). The profile is similar to that of the simulation, and the data have been fitted to this dependence. They are consistent with a lower p*K*_a_ of 6.5, and a higher p*K*_a_ > 9 that cannot be measured due to the instability of RNA at high pH. The initial rise in activity over the range pH = 6.0–7.5 shows that the reaction rate requires the deprotonated form of a catalytic component; this could be a general base, but would also be consistent with deprotonation of the nucleophile or solvent water, for example. This increase in reaction rate at the lower pH levels off to form a plateau, indicating the requirement of a protonated component with an apparent p*K*_a_ of 6.6. This would be consistent with the role of C57 as a general acid as suggested by its position in the crystal structures, and a closely similar pH dependence and p*K*_a_ was found for the viral HDV ribozyme by Bevilacqua and coworkers^31^. The p*K*_a_ > 9 would be consistent with a role of a hydrated metal ion as a general base, or perhaps deprotonation of solvent water that could act as a specific base. Although formally we cannot exclude the lower and higher p*K*_a_ values arising from general base and acid, respectively (an example of kinetic ambiguity), this would be very much less probable in the light of the structural data.

### Mutation of C57, the putative general acid

**Fig. 5:**
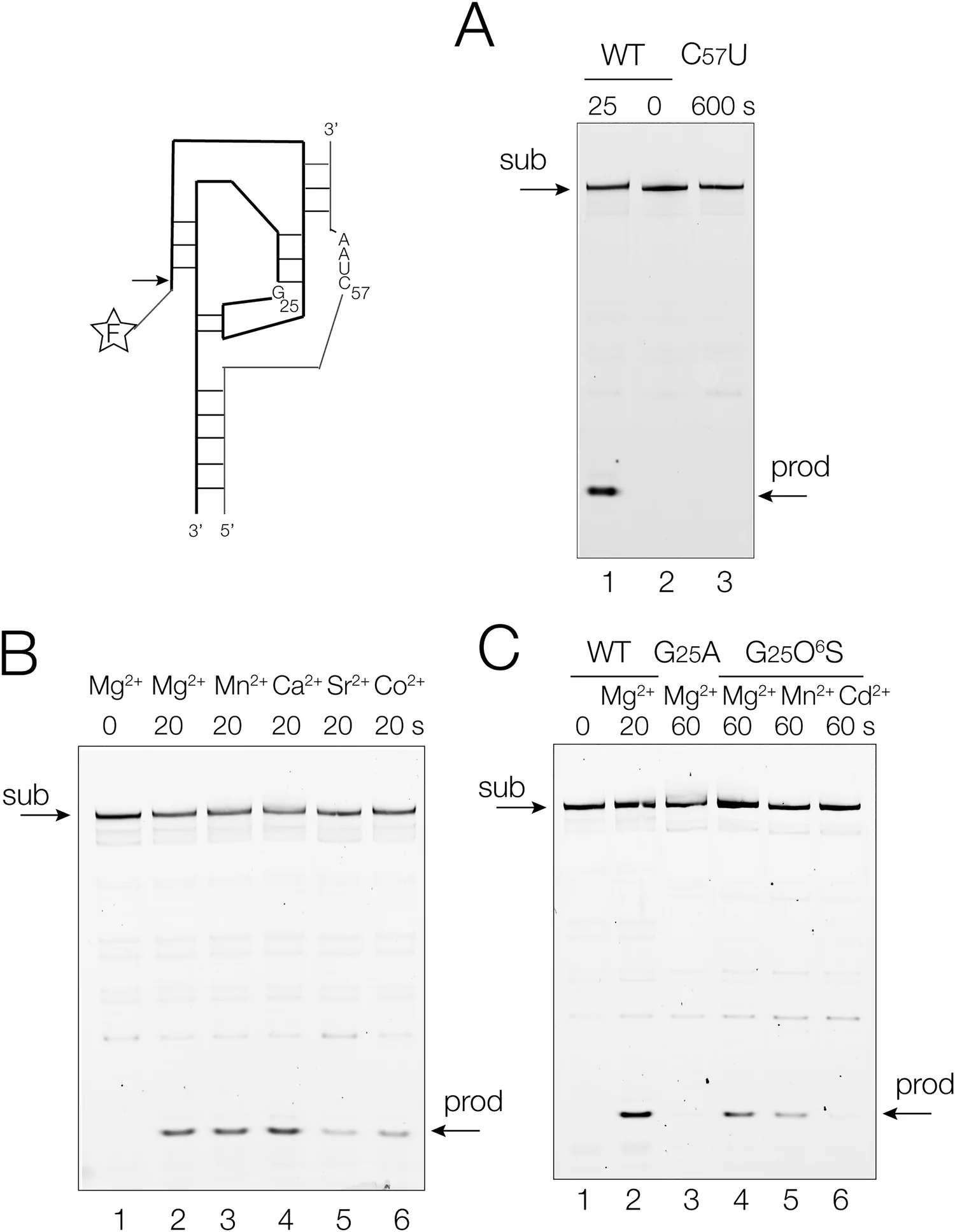
The effect of mutation and metal ions on the cleavage activity of the *C*. *briggsae* HDV ribozyme. 5’-fluorescein-labeled RNA (scheme top left) was incubated under single-turnover conditions, and the substrate (*sub*) and cleaved product (*prod*) were separated by polyacrylamide gel electrophoresis under denaturing conditions. **A** The effect of a C57U mutation on cleavage activity. Wild-type sequences were incubated for 0 and 25 s (tracks 2 and 1, respectively). Incubation with C57U ribozyme (track 3) gave no detectable cleavage after 10 min. incubation. **B** Activity in different divalent metal ions. Ribozyme and substrate were incubated under standard conditions that included 2 mM of Mg^2+^, Mn^2+^, Ca^2+^, Sr^2+^ or Co^2+^ for 20 s (tracks 2–6, respectively). RNA was incubated for zero time in track 1. The rates of cleavage in Mn^2+^, Ca^2+^ (Supplementary Fig. S10) and Na^+^ ions (Supplementary Fig. S11) have been measured under single turnover conditions. **C** Effect of mutation of a putative metal ion binding site at G25. Tracks 1 and 2 respectively: incubation of wild-type RNA under standard conditions that included 2 mM of Mg^2+^ for 0 and 20 s. Track 3: incubation of G25A RNA under standard conditions, including 2 mM of Mg^2+^ for 60 s. Tracks 4–6: incubation of ribozyme containing a G25O^6^S atomic mutation under standard conditions, including 2 mM of Mg^2+^ (track 4), Mn^2+^ (track 5) or Cd^2+^ (track 6). We have measured the rate of cleavage of G25O^6^S in the presence of 2 mM of Mg^2+^ (Supplementary Fig. S12).

The position of the nucleobase of C57 in the *C. briggsae* ribozyme (and that of C58 in the *Ackermannviridae* ribozyme) observed in the crystal structure (Fig. 3D) is consistent with a role as a general acid to protonate the O5’ leaving group in the cleavage reaction, as has been shown for the viral HDV ribozyme^29^. It is also consistent with the observed p*K*_a_ of 6.*5* determined above. We therefore tested this by examining the cleavage activity of a C57U mutant of the *C. briggsae* ribozyme, where the imino proton attached to N1 of the uridine is not acidic. In contrast to the wild-type sequence ribozyme, the C57U mutant exhibited no detectable ribozyme cleavage activity even after prolonged incubation (Fig. 5A). No activity was also observed for the *C. briggsae* HDV C57U ribozyme divided at the J1/2 connection (Fig. 2). In addition, we also examined the cleavage activity of the *Ackermannviridae* ribozyme with the corresponding C58U mutation. This, too, exhibited undetectable levels of activity after prolonged incubation (Supplementary Fig. S9). These results are consistent with the proposed role of the C57 nucleobase as a general acid.

### Activity in different metal ions

In the pre-cleavage crystal structures of the HDV ribozyme, we observe a divalent metal ion bound into the active center (Fig. 3D). A hydrated metal ion has been proposed as a candidate for the general base in the cleavage reaction^31^, or it might also play a role as a Lewis acid. While the majority of the experiments described here have been performed in the presence of magnesium ions, we also investigated cleavage activity in other divalent metal ions. The *C. briggsae* HDV ribozyme was incubated in 20 mM Tris.HCl (pH 7.5) containing 2 mM Mg^2+^, Mn^2+^, Ca^2+^, Co^2+^ or Sr^2+^ ions for 20 s, and substrate and product separated by gel electrophoresis under denaturing conditions (Fig. 5B). Inspection of the gel suggests that incubation in the presence of Mg^2+^, Mn^2+^ or Ca^2+^ ions leads to similar levels of cleavage activity. Lower rates of cleavage are apparent in the presence of Co^2+^ and Sr^2+^ ions. The rates of cleavage in Mn^2+^ and Ca^2+^ ions were measured (Supplementary Fig. S10), and together with that in Mg^2+^ ions, the rates are all within a factor of 2, in the range of 6.7–11.1 min^−1^ (Table 1), in the order Ca^2+^ > Mg^2+^ > Mn^2+^. We also tested the cleavage activity of the *C. briggsae* HDV ribozyme in a high concentration of monovalent metal ions. We observed significant cleavage of the substrate RNA in the presence of 2 M Na^+^ ions, but with a rate of 0.01 min^−1^ (Supplementary Fig. 11), i.e., 1000-fold slower than in the above divalent metal ions.

### Mutation of G25, a potential metal ion binding site

We observe a metal ion bound in the active center of the HDV ribozyme pre-cleavage structure. However, since there was no 2’-hydroxyl group at the −1 position in the crystalized ribozyme (in order to remove the nucleophile and thus prevent attack on the scissile phosphate), the position of the metal ion could be displaced from its position in the active structure. Furthermore, as discussed below, our observed structure is not in the conformation required for in-line attack, and adjustment into a geometry consistent with activity would likely lead to repositioning of the metal ion. In the viral HDV ribozyme Golden and coworkers^28,30^ have observed a divalent metal ion bound via its inner hydration sphere to O6 and N7 of G25 that forms a G:U base pair with U20. Our observed metal ion is located >5 Å from G25 O6 and N7. We hypothesized that the metal ion might become bound to G25 in the active structure where the nucleophilic O2’ is present, and therefore examined whether or not cleavage activity might be affected by mutation at G25. We found that a G25A mutation led to a marked reduction in ribozyme cleavage (Fig. 5C, track 3), with no observable cleavage after 60 s incubation. We then tested an atomic mutation in which G25 was replaced by 6-thioguanine, i.e., sulfur was substituted in place of G25 O6 (G25 O^6^S). This also led to significant impairment of ribozyme cleavage activity (Fig. 5C, track 4). We measured a rate of cleavage corresponding to 0.010 ± 0.001 min^−1^ for this variant (Supplementary Fig. S12), corresponding to a 1000-fold reduction in cleavage activity.

If direct binding of a metal ion was required at G25 O6 then replacement by sulfur might weaken binding, but some activity might be restored by incubation in the presence of a softer, more thiophilic metal ion. We therefore performed further incubations of the G25 O6 ribozyme in the presence of manganese (II) or cadmium (II) ions. However, no restoration of ribozyme activity was observed on replacing 2 mM Mg^2+^ either with Mn^2+^ ions or Cd^2+^ ions (Fig. 5C tracks 5 and 6, respectively).

## DISCUSSION

HDV-like ribozyme sequences have been identified in new organisms, including invertebrate species. These are very active, with rates of cleavage of 4 and 9 min^−1^, corresponding to an acceleration of cleavage of approximately a million-fold. This places the HDV ribozymes amongst the fastest of the group of nucleolytic ribozymes, comparable with the pistol and pseudoknot-containing hammerhead ribozymes^41^. We note that the fastest ribozymes in this class are all based upon pseudoknot-containing structures, perhaps indicating that this is a particularly stable way to achieve an active fold of the RNA. In the course of this investigation we found that the HDV ribozymes can be very sensitive to the integrity of the J1/2 linker in some cases, and this is likely to result from a lowered stability of the P1.1 helix. It is therefore a safer strategy to open the P4 helix to convert it into a two-piece ribozyme-plus-substrate construct.

The activity of both cellular and viral ribozymes has been retained through evolution, suggesting an important function of these ribozymes. These can now be added to the original HDV viral enzyme (for which the function in processing replication intermediates is established) and the human ribozyme CPEB1^21,22^ and related orthologs^21^. Unfortunately no function has been ascribed to the cellular HDV ribozymes yet, but we feel this must exist. The sequences of these ribozymes confer high activity; they have not acquired mutations disabling catalytic activity pointing to a selective advantage for preservation of activity.

Our measured pH dependence of the cleavage activity of the *C. briggsae* HDV ribozyme follows a log-linear rise in activity at lower pH that levels off and reaches a plateau at pH 8 (Fig. 4C). The data fit an apparent p*K*_a_ = 6.5, and a second significantly higher p*K*_a_. This is very similar to the pH dependence measured by Bevilacqua and colleagues for the viral HDV ribozyme^31^. In general terms, this indicates that the activity of the ribozyme requires deprotonation of some element (the general rise in activity up to pH 7), and protonation of another (the leveling off in activity at pH 7). In principle, these could be a general base and an acid, respectively. We confidently assign the lower p*K*_a_ to C57 acting as general acid to protonate the O5’ oxyanion leaving group in the cleavage reaction. This is consistent with the C57U mutant exhibiting no detectable activity (Fig. 5A) (and the corresponding C58U in the *Ackermannviridae* HDV ribozyme (Fig. 2)). It is also supported by the position of C57 in the active center of the *C. briggsae* HDV ribozyme, both before and after cleavage, that of C58 in the *Ackermannviridae* HDV ribozyme post cleavage (Fig. 3), and in that of the viral HDV ribozyme^29^. The role of the general acid in the HDV ribozyme is well established, and this is clearly a general mechanism in this class of nucleolytic ribozyme. The origin of the higher (undetermined) p*K*_a_ has not been determined with confidence. In principle, this might be due to deprotonation of a general base required to deprotonate the O2’ nucleophile; we return to this below.

**Fig. 6:**
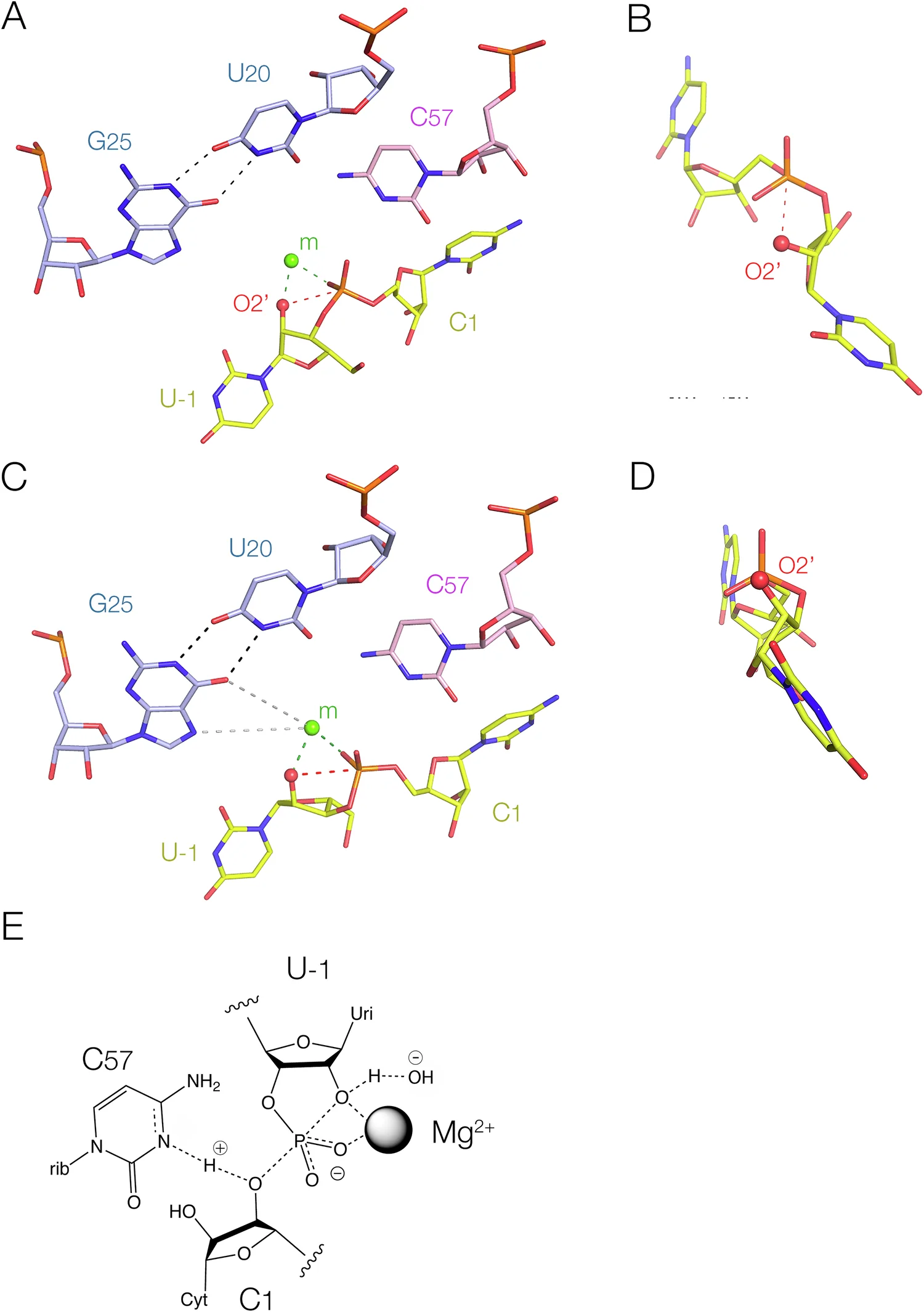
Remodeling the active center of the HDV ribozyme, and the deduced catalytic mechanism. **A**,**B** The active center of the *C. briggsae* HDV ribozyme with an oxygen atom modeled at the U-1 2’ position, with nothing else altered. The red broken line connects the O2’ and P atoms, representing the direction of nucleophilic attack. The bound metal ion observed in the crystal structure is shown by the green sphere. This lies 2.2 Å from the modeled O2’ atom and the *pro*-R non-bridging O atom (green broken lines). **A** The overall view. **B** A view down the axis of the P – O5’ bond showing the deviation from in-line geometry. **C**, **D** The active center of the *C. briggsae* HDV ribozyme remodeled to achieve a closer to in-line geometry, and a slightly altered metal ion position. **C** The overall view. The metal ion lies 2.3 Å from the O2’ atom, 2.2 Å from the *pro*-R non-bridging O atom, 4.7 Å from G25 O6 and 5.2 Å from G25 N7. **D** A view down the axis of the P – O5’ bond showing that the remodeled structure is close to an in-line geometry. **E** The proposed catalytic mechanism of the *C. briggsae* and *Ackermannviridae* HDV ribozymes, consistent with the structural and mechanistic data. The catalytic rate enhancement arises from a combination of metal ion-mediated Lewis acid activation of the nucleophilic attack, and general acid catalysis by C57 (C58 in the *Ackermannviridae* HDV ribozyme) protonating the O5’ oxyanion leaving group.

In the active center of the pre-cleavage structure of the *C. briggsae* HDV ribozyme, we observe both the U-1 and C1 nucleotides, a bound Mg^2+^ ion, and the C57 nucleobase that is the likely general acid (Fig. 3D). Our pre-cleavage structure was obtained by incorporation of a 2’-deoxynucleotide at the −1 position, so removing the potential nucleophile. When we modeled the O2’ atom into our pre-cleavage structure (Fig. 6A), we found that its position deviated significantly from that required for an in-line attack, although the O2’-P distance was optimal, at 3 Å. The local conformation is a result of a base pair formed between U-1 with an adenine in a symmetry-related molecule in the crystal lattice (Supplementary Fig. S7). As a result of this lattice contact, the measured O2’ - P - O5’ angle was 131°, i.e. 49° away from in-line attack, giving a Breaker in-line fitness parameter^42^ of 0.58 (Fig. 6B). We therefore performed a minimal manual adjustment of the local conformation, with the largest change being a 117° rotation in the torsion angle about the O3’ – C3’ bond and a smaller rotation of 39° in that about the P – O3’ bond. The conformational adjustment was straightforward with a path that encountered no intermediate steric clash. The resulting structure (Fig. 6C) has an O2’ - P - O5’ angle of 172° (approximately a *g*- to *g*+ conformational change) with a corresponding in-line fitness parameter of 0.94 (Fig. 6D). Thus, the potentially active conformation of the ribozyme is fully accessible from our observed structure.

In the active center, there is a strong peak of electron density corresponding to a Mg^2+^ ion bound 2 Å from the *pro*-R non-bridging oxygen atom of the scissile phosphate (Supplementary Fig. S8). The activity of the new HDV ribozymes depends on the presence of metal ions. Divalent metal ions (especially Mg^2+^, Mn^2+^ or Ca^2+^) lead to high rates of cleavage (Fig. 5B and Table 1). Moreover, the activity in the presence of 2 mM Mg^2+^ ions is only diminished by concentrations of the trivalent ion Co(III) hexammine of 10 mM or greater (unpublished data) indicating that the Mg^2+^ ion is very tightly bound into the active center, although greater inhibition of cleavage by the trivalent ion was observed for the viral HDV ribozyme^31^. Furthermore, Bevilacqua and colleagues^34^ have observed a stereospecific effect of sulfur substitution for the *pro*-R non-bridging oxygen atom of the scissile phosphate for the viral HDV ribozyme. Collectively, these data indicate that the Mg^2+^ ion is directly bound to the phosphate oxygen atom. This in turn suggests that it could act as a Lewis acid catalyst, polarizing the P-O bond to increase the electrophilicity of the phosphorus atom and thus enhancing nucleophilic attack by the O2’ atom. Golden and co-workers^30,35^ have similarly proposed that the viral HDV ribozyme uses Mg^2+^-ion mediated Lewis acid catalysis of the cleavage reaction. In addition, they have observed binding of their active site Mg^2+^ ion to O6 and N7 of G25 that forms a reverse-wobble base pair with U20 adjacent to the active center^28,30^. Recently, Piccirilli and co-workers have proposed a mechanism whereby the Mg^2+^ ion directly coordinates both the *pro*-R non-bridging oxygen and the O2’ atom in the transition state based on kinetic isotope effects and QM/MM calculations^36^. In the *C. briggsae* HDV ribozyme we found that G25A mutation lead to marked impairment of cleavage activity (Fig. 5C). Furthermore, the atomic mutation of G25 O6 to sulfur resulted in a 1000-fold reduction in cleavage rate (Supplementary Fig. S12). That change could not be compensated by changing to a softer metal ion, either Mn^2+^ or Cd^2+^ (Fig. 5C). It is therefore unlikely that the Mg^2+^ ion is directly bound to O6, and is more likely to make a water-mediated interaction via its inner sphere of hydration. We therefore made a further small adjustment in the position of the metal ion in our re-modeled active center so that it is now located 4.7 Å from G25 O6 and 5.3 Å from G25 N7 (consistent with water-mediated interactions) and 2.2 Å (direct bonding) to the O2’ nucleophile and the *pro*-R non-bridging oxygen atom of the scissile phosphate (Fig. 6C). This model involved very minor changes to the structure observed in the crystal, and is consistent with all the structural and experimental mechanistic data.

We think that the collective evidence for direct binding of a Mg^2+^ ion to the scissile phosphate is strong, and therefore that its role in Lewis acid catalysis is very probable. The evidence for general acid catalysis, protonating the O5’ leaving group, is also strong. That leaves two (probably related) questions. What removes the proton from the O2’ nucleophile, and what is responsible for the rise in ribozyme activity with pH below neutrality? At the present time, we do not have a definitive answer to this. The structure does not suggest participation by an RNA component acting as a general base. Nakano et al.^31^ proposed that a hydrated divalent metal ion acts as a general base. In the catalytic mechanism of the TS ribozyme, the available evidence points to a bound hydrated Mg^2+^ ion acting as the general base to deprotonate the O2’ nucleophile. This results in a log-linear dependence of the rate on the p*K*_a_ of the water of hydration of the metal ion with unit gradient^43^ (Supplementary Fig. S13). Similarly, the pistol ribozyme exhibits a log-linear dependence of cleavage rate of metal ion p*K*_a_ with unit gradient^44^—in this case, the evidence indicates that a bound Mg^2+^ ion acts as the general acid to protonate the O5’ oxyanion leaving group. The complete absence of a correlation of activity with p*K*_a_ for the HDV ribozymes suggests that while the metal ion is likely playing a role in catalysis, this does not involve proton transfer from its inner hydration sphere. It remains possible that there could be an alternative explanation for these data. But for the TS ribozyme, the dependence of cleavage rate upon p*K*a is exactly what would be predicted for ion-mediated general base catalysis, in contrast to what we observe for the HDV ribozyme here. That leaves the solvent water as a potential participant. If water itself acted to accept the proton, this should be pH-independent. That would mean that the reaction rate should follow *f*_A_ (the fraction of protonated general acid) alone, i.e., be the opposite of what we observe. Therefore, it seems more likely that hydroxide might act as a specific base. Its concentration will be low at neutrality, but in combination with Lewis acid catalysis by the metal ion this may be sufficient.

The minimally remodeled structure of the *C. briggsae* HDV ribozyme structure is consistent with all the mechanistic data, notably the reaction pH dependence, the absence of a log-linear dependence upon metal ion p*K*_a_, and mutagenesis. This indicates that the new HDV ribozymes use a catalytic mechanism involving general acid catalysis to protonate the 5’-oxyanion leaving group and divalent metal ion-mediated Lewis acid catalysis to activate nucleophilic attack by the O2’ atom (Fig. 6E). The results are consistent with earlier analysis of the viral HDV ribozyme mechanism, suggesting that both structure and mechanism are well conserved in this class of ribozyme. The proposed role for metal ion-mediated Lewis acid activation of nucleophilic attack is a significant difference from other nucleolytic ribozymes that use general base catalysis. It also contrasts with those ribozymes that use hydrated metal ions as general base (TS^43^) or a general acid (pistol^44^ and hammerhead^44,45^). The catalytic use of a metal ion as a Lewis acid implies that mechanistically the HDV ribozyme is intermediate in character between the nucleolytic ribozymes that use general acid-base catalysis and larger ribozymes such as the group I intron ribozymes that principally use metal ions to organize their transition states and activate catalysis^10–15,46^.

## METHODS

### Preparation of RNA

RNA sequences for analysis of cleavage are tabulated in Supplementary Table S1. Both strands of the *C. briggsae* and *Ackermannviridae* HDV-like ribozymes, divided at the P4 stem-loop, and the shorter (substrate) strands, divided at the J1/2 connection, were generated by chemical synthesis. RNA was synthesized using *t*-BDMS phosphoramidite chemistry implemented on Applied Biosystems 394DNA/RNA synthesizers as described in Wilson et al.^47^, followed by standard deprotection and desilylation procedures^48^. The longer (ribozyme) strands of both ribozymes, divided at the J1/2 connection, were generated by T7 RNA polymerase transcription from a DNA template generated by recursive PCR from synthetic DNA oligonucleotides using Q5 DNA polymerase (Master Mix, NEB). Substrate RNA in J1/2-divided RNA was radioactively [5’-^32^P]-labeled using T4 polynucleotide kinase. Substrate RNA in P4-divided RNA was 5’-fluorescently labeled with fluorescein (*C. briggsae*) or Cy-3 (*Ackermannviridae*). All RNA was purified by gel electrophoresis under denaturing conditions and recovered by passive diffusion.

RNA for crystallization was prepared by in vitro transcription from PCR-amplified DNA templates using T7 RNA polymerase. The resulting RNA molecules were purified by denaturing polyacrylamide gel electrophoresis (7 M urea). Full-length transcripts were visualized by ultraviolet shadowing, excised, and electroeluted using the Elutrap Electroelution System (GE Healthcare) in 0.5× Tris-borate-EDTA (TBE) buffer for 12 h at 200 V at 4 °C. The RNA was then precipitated with isopropanol, washed with 75% ethanol, and resuspended in RNase-free water. Pre-cleavage HDV ribozyme RNA was purchased commercially: the short-chain section from Accurate Biotechnology (Hunan, China) Co., Ltd., and the long-chain portion from Weihuan Biotechnology (Shanghai, China) Co., Ltd., with the 5′ terminal uridine substituted with deoxyuridine (dU) to prevent self-cleavage. All DNA templates and RNA sequences used in this study are listed in Supplementary Table S2.

### Ribozyme kinetics

Standard conditions for studying ribozyme cleavage were single-turnover conditions in 20 mM Tris.HCl (pH 7.5), 2 mM MgCl_2_ at 37 °C unless otherwise stated. Reactions at pH 6.0 and 6.7 were studied in 20 mM MES buffer, and reactions at pH 8.5 were studied in 20 mM Tris.HCl. The pH of the reaction buffers was measured using an Accumet AB150 pH meter (Fischer Scientific). Reactions were also studied where the divalent metal ion was replaced by 2 mM Mn^2+^, Ca^2+^, Sr^2+^, Co^2+^or Cd^2+^, or 2 M Na^+^.

150 nM labeled RNA was hybridized with 1 µM unlabeled RNA by heating to 80 °C then cooling to 25 °C at 0.2 °C per second in a thermal cycler. After preincubation at 37 °C, ribozyme cleavage was initiated by the addition of MgCl_2_ to a final concentration of 2 mM. Fast reactions were studied by mixing 2 µl droplets containing RNA and metal ions for 1–60 s on a hydrophobic surface, then mixing with three volumes of 50 mM EDTA in formamide. Slower reactions were measured in plastic tubes and aliquots were removed at chosen times and cleavage terminated by addition of 3 volumes of 50 mM EDTA in formamide. Slow reactions requiring incubations > 1 h were carried out under mineral oil to prevent evaporation. Substrate and product RNA were separated by electrophoresis in 30 cm 20% polyacrylamide gels under denaturing conditions, and imaged by autoradiography (^32^P) or fluorography (fluorescein or Cy3) using a Typhoon FLA 9500 fluorimager (GE Healthcare). Band intensities were quantified using ImageQuant, plotted and fitted by non-linear regression analysis to single exponential functions using KaleidaGraph (Abelbeck Software) to calculate rate constants.

### Statistics and reproducibility

All kinetic rate constants have been calculated from ≥3 independent measurements, and presented as mean ± standard deviation. The number of replicates for each rate is shown in Table 1. In all cases, rate constants were calculated from the dependence of reaction progress on time for independently prepared reaction mixes. In addition, mean values and standard deviations could be calculated for each time point where rates were measured by mixing droplets.

### Simulation of pH dependence of cleavage rate

The observed rate of cleavage (*k*_obs_) will be lower than the intrinsic rate when the ribozyme is fully active (*k*_cat_) according to:

1 $${k}_{{obs}}={k}_{{cat}}\cdot {f}_{A}\cdot {f}_{B}$$

where *f*_A_ is the fraction of protonated acid, and *f*_B_ is the fraction of deprotonated base. The fractions *f*_A_ and *f*_B_ at any given pH are calculated as^49^:

2 $${f}_{A}=\left\{1+{10}^{\left(p{K}_{a}^{B}-{pH}\right)}\right\}/\left\{1+{10}^{\left(p{K}_{a}^{B}-{pH}\right)}+{10}^{\left(p{K}_{a}^{B}-p{K}_{a}^{A}\right)}+{10}^{\left({pH}-p{K}_{a}^{A}\right)}\right\}$$

3 $${f}_{B}=\left\{1+{10}^{\left({pH}-p{K}_{a}^{A}\right)}\right\}/\left\{1+{10}^{\left(p{K}_{a}^{B}-{pH}\right)}+{10}^{\left(p{K}_{a}^{B}-p{K}_{a}^{A}\right)}+{10}^{\left({pH}-p{K}_{a}^{A}\right)}\right\}$$

where the p*K*_a_ values of the general acid and base are written $p{K}_{a}^{A}$ and $p{K}_{a}^{B}$ respectively.

### Crystallization and structure determination by X-ray crystallography

Solutions containing 10 μg/μl RNA were prepared in a buffer consisting of 5 mM HEPES (pH 7.5), 100 mM KCl, and 5 mM MgCl₂ and were denatured at 95 °C for 1 min. Crystallization was achieved using the hanging-drop vapor diffusion method by mixing 0.8 μl RNA solution with 0.8 μl reservoir solution at 18 °C. RNA sequences and crystallization conditions are summarized in Supplementary Tables S2 and S3. High-quality crystals were mounted directly on nylon loops and flash-frozen in liquid nitrogen for data collection.

X-ray diffraction data were collected at the Shanghai Synchrotron Radiation Facility (SSRF) on beamlines BL02U1 or BL10U2 and were processed using XDS^50^. The resolution cutoff for the data was determined by examining the CC1/2 and the electron density map^51^. The structure of the cleaved C. *briggsae* HDV ribozyme deposited as 22KS was determined by molecular replacement using PHASER^52^ with the previously reported HDV ribozyme structure (PDB 1DRZ)^26^ as the search model. The resulting structure (PDB 22KS) was subsequently used as the molecular replacement template to determine the structures of the pre-cleavage C. *briggsae* HDV ribozyme (PDB 22KX) and the *Ackermannviridae* HDV ribozyme (PDB 22KW).

All models were manually adjusted using Coot^53^ and refined through iterative cycles of refinement using Phenix.refine^54^ and PDB-REDO^55^. Model geometry and agreement with electron-density maps were evaluated using MolProbity^56^ and the validation tools implemented in Coot. Simulated annealing omit maps were calculated using the composite omit map procedure in the PHENIX suite with the anneal protocol. Atomic coordinates and structure factor amplitudes have been deposited in the Protein Data Bank^57^ under the accession codes listed in Supplementary Table S4, together with data collection and refinement statistics.

### Reporting summary

Further information on research design is available in the Nature Portfolio Reporting Summary linked to this article.

## Supplementary information

Supplementary Information

Reporting Summary

Transparent Peer Review file

## Source data

Source Data

## Data Availability

The data supporting the findings of this study are available from the corresponding authors upon request. The coordinates and structure factors of all reported crystal structures have been deposited in the PDB under the following accession numbers: 22KX (HDV-like ribozyme from *C. briggsae*, pre-cleaved state); 22KS (HDV-like ribozyme from *C. briggsae*, cleaved state); and 22KW (HDV ribozyme from *Ackermannviridae*, cleaved state). Source data for the figures and Supplementary Figs. are provided as a Source Data file. Source data are provided with this paper.
