# Supplementary material for: Structure and mechanism of HDV ribozymes revealing Lewis acid catalysis by a metal ion
Source: Nat Commun. 2026 Sep 2;17:10462. doi: 10.1038/s41467-026-77452-w (PMC13635239; doi:10.1038/s41467-026-77452-w)
Supplement: Supplementary file 1 [file 41467_2026_77452_MOESM1_ESM.pdf]

Structure and mechanism of HDV ribozymes revealing Lewis acid catalysis by a metal ion

Yuhang Luo, Xiaoxue Chen, Xiaowei Lin, Wenjian Liao, Bowen Xiao, Mengxiao Li, Zhaoji Qiu, Timothy J. Wilson, Miao Zhichao, Jia Wang, Lin Huang and David M. J. Lilley

SUPPLEMENTARY INFORMATION

3 August 2026

SUPPLEMENTARY FIGURES

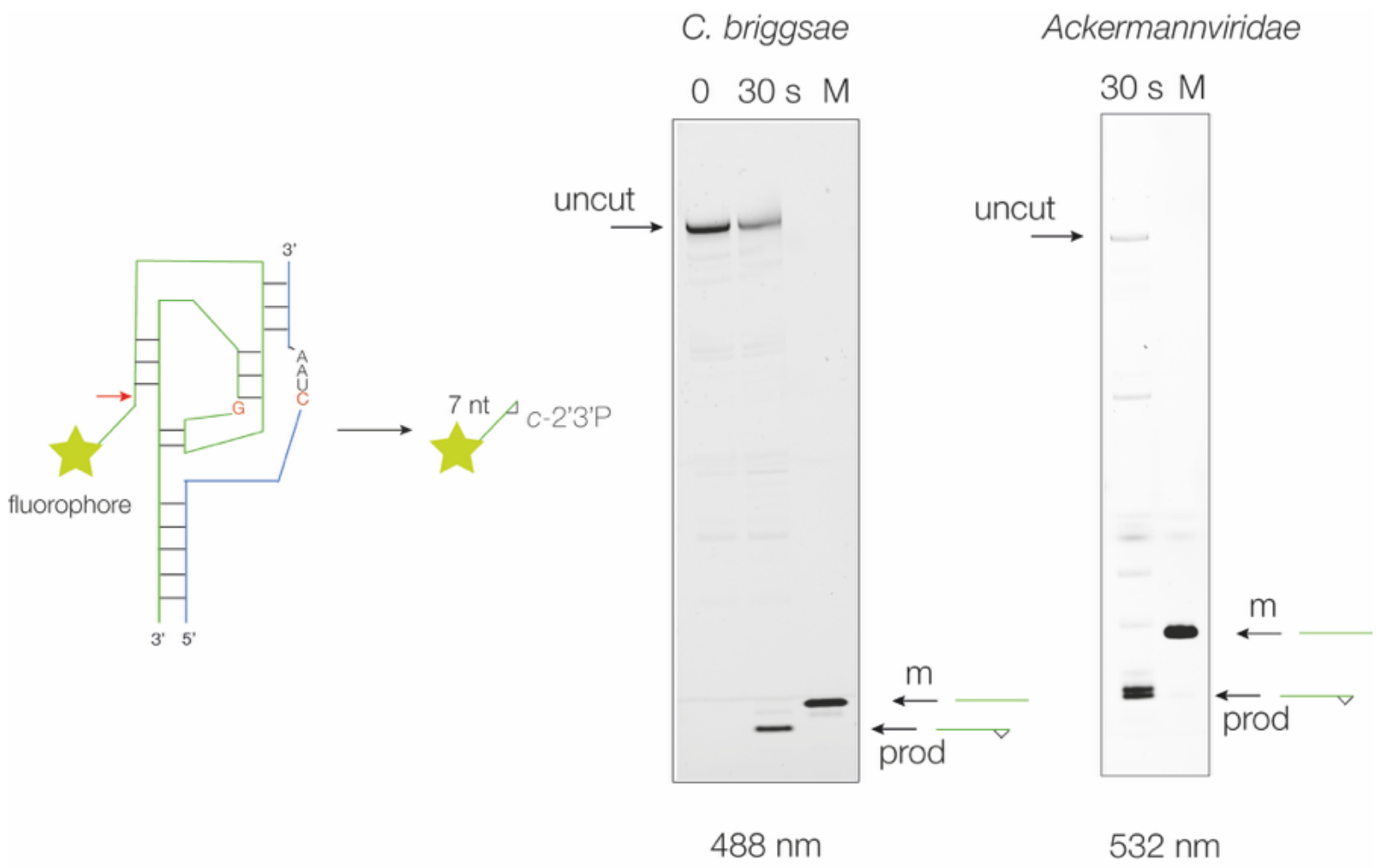

Supplementary Figure S1. Site-specific cleavage by the *C. briggsae* and *Ackermannviridae* HDV ribozymes. These reactions were performed using ribozymes divided at the loop of the P4 helix (as shown on the left) and fluorescently-labeled with fluorescein and Cy3 respectively at the 5' terminus. Ribozyme cleavage was carried out for 30 s under our standard conditions, and full-length and product RNA separated by electrophoresis in 30 cm 20 % polyacrylamide gels under denaturing conditions. Fluorescein and Cy3 were excited at 488 and 532 nm respectively. 7 nt RNA species having the sequence of the expected products [*C. briggsae* (fluorescein-CCAUUUU) and *Ackermannviridae* (Cy3-GCAUCCA)] were generated by chemical synthesis and used as a guide to the mobility of the product of ribozyme cleavage. Since ribozyme cleavage transfers the phosphate to the 3' end of the RNA as a cyclic 2'3' phosphate, the products have an additional charged phosphate group compared to the synthetic oligonucleotides, and are therefore expected to migrate fractionally faster. We observe that for both ribozymes, the product of cleavage migrates a little faster than the marker oligonucleotide.

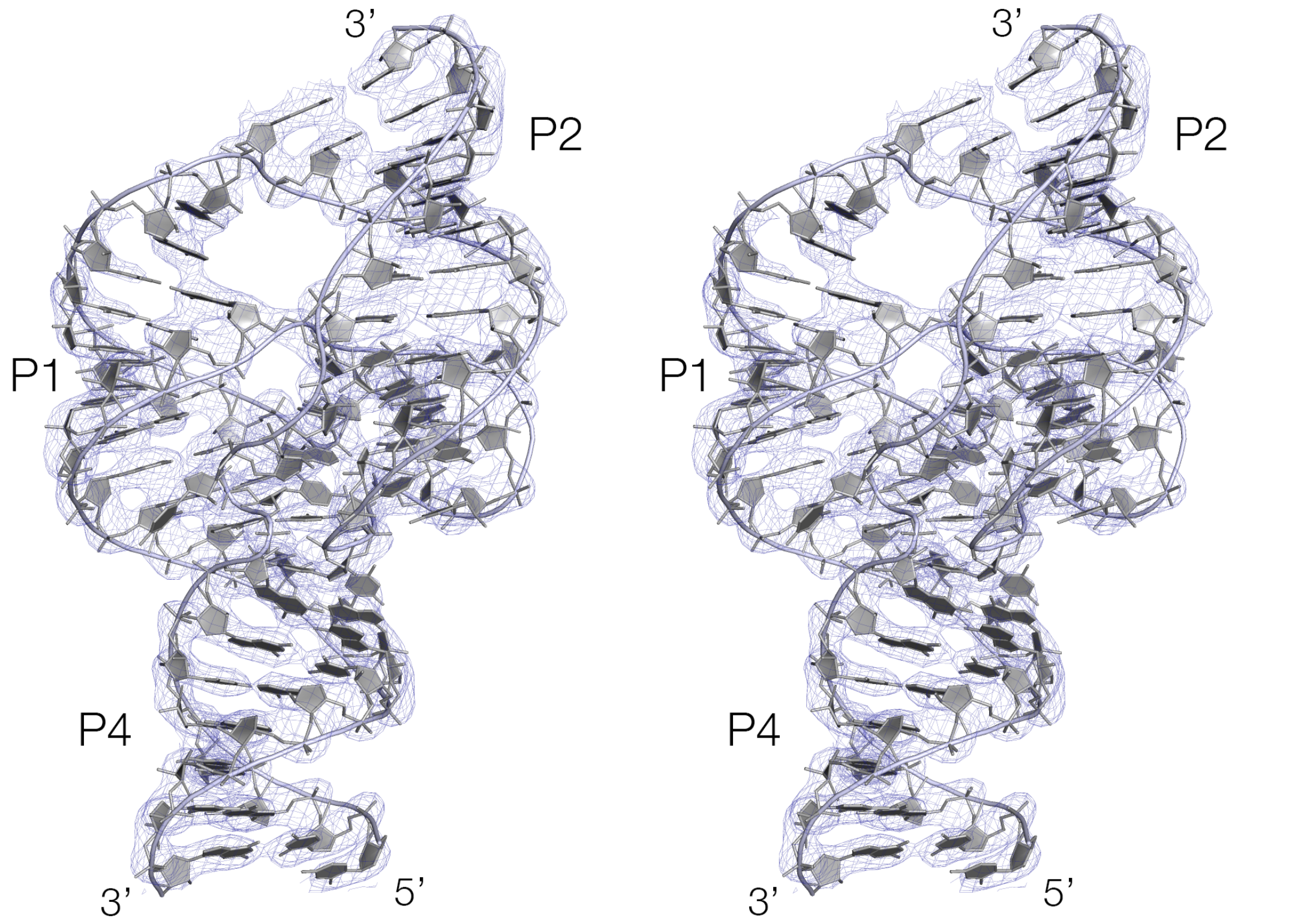

Supplementary Figure S2. The crystal structure of *C.briggsae* HDV ribozyme as a pre-cleavage complex showing the electron density map contoured at 1.5 σ drawn as a parallel-eye stereoscopic image.

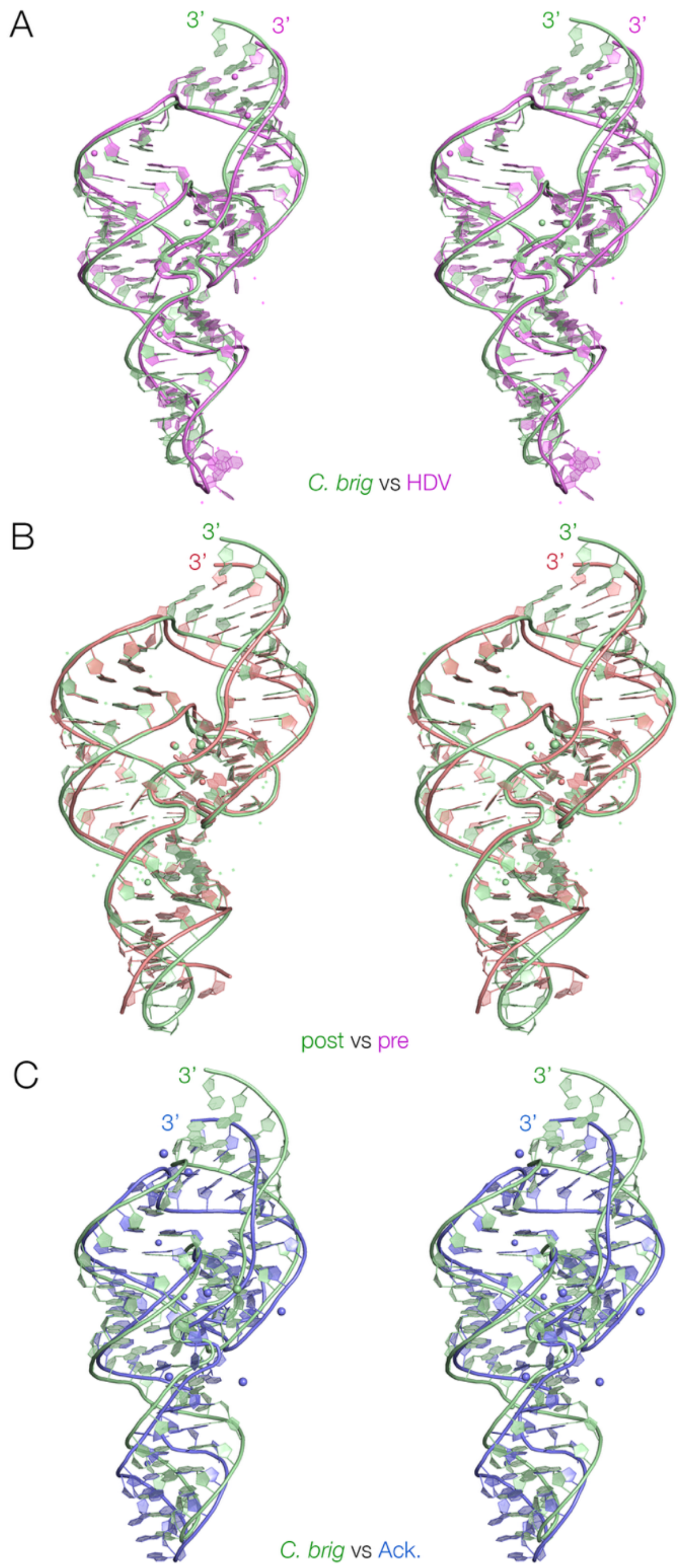

Supplementary Figure S3. Superposition of HDV structures.

A. Superposition of *C. briggsae* post-cleavage ribozyme structure (green) with the structure of the viral HDV ribozyme (violet; PDB ID 1DRZ).

B. Superposition of *C. briggsae* post- (green) and pre-cleavage (red) ribozyme structures.

C. Superposition of *C. briggsae* (green) and the *Ackermannviridae* (blue) post-cleavage ribozyme structures.

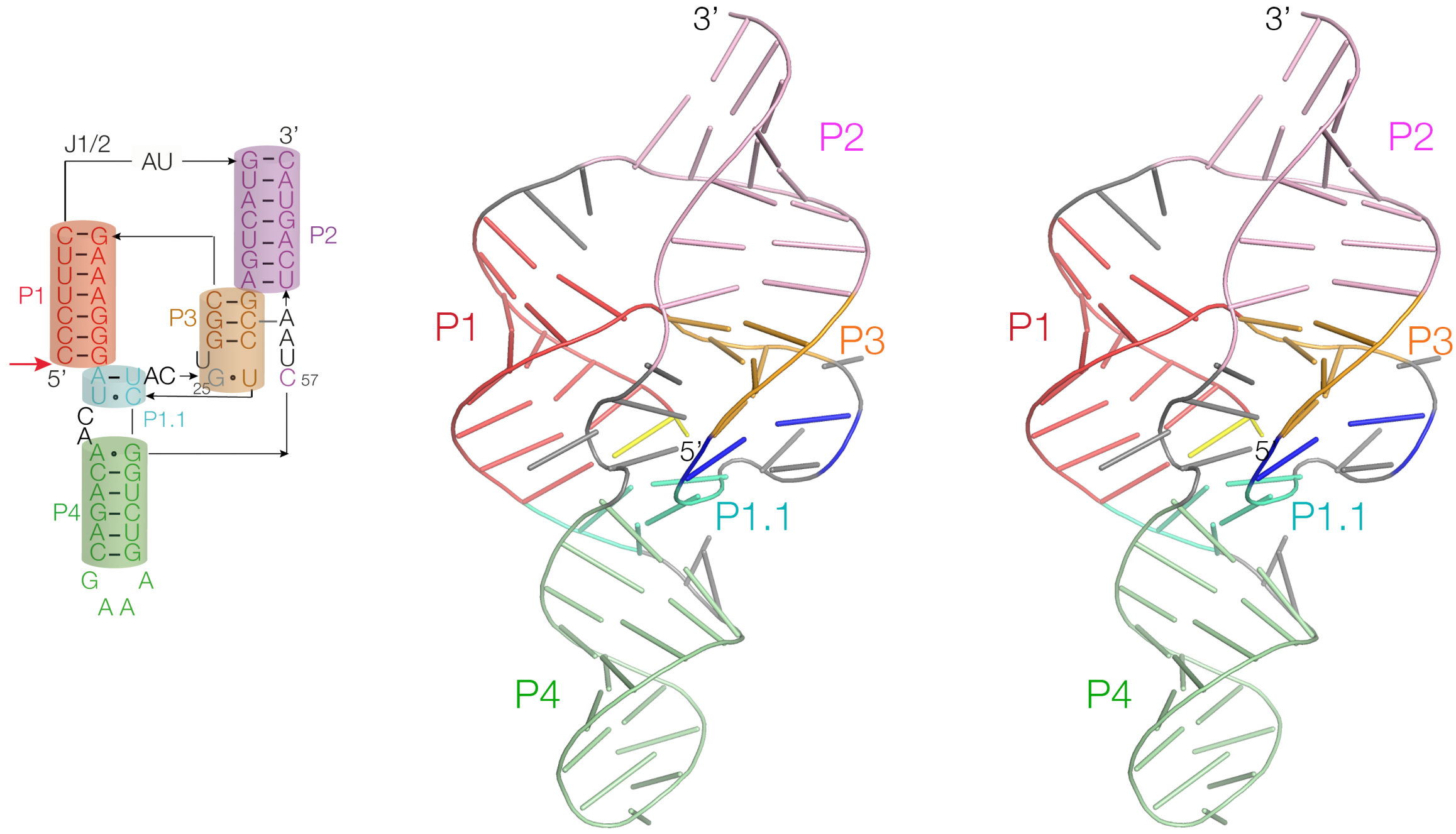

Supplementary Figure S4. The crystal structure of *C.briggsae* HDV ribozyme post-cleavage complex drawn as a parallel-eye stereoscopic image. The structure is depicted in cartoon mode. The 5'-terminal G1 is colored yellow and the U20:G25 basepair is colored blue. Note that the active center of the ribozyme is shown in Figure 3E.

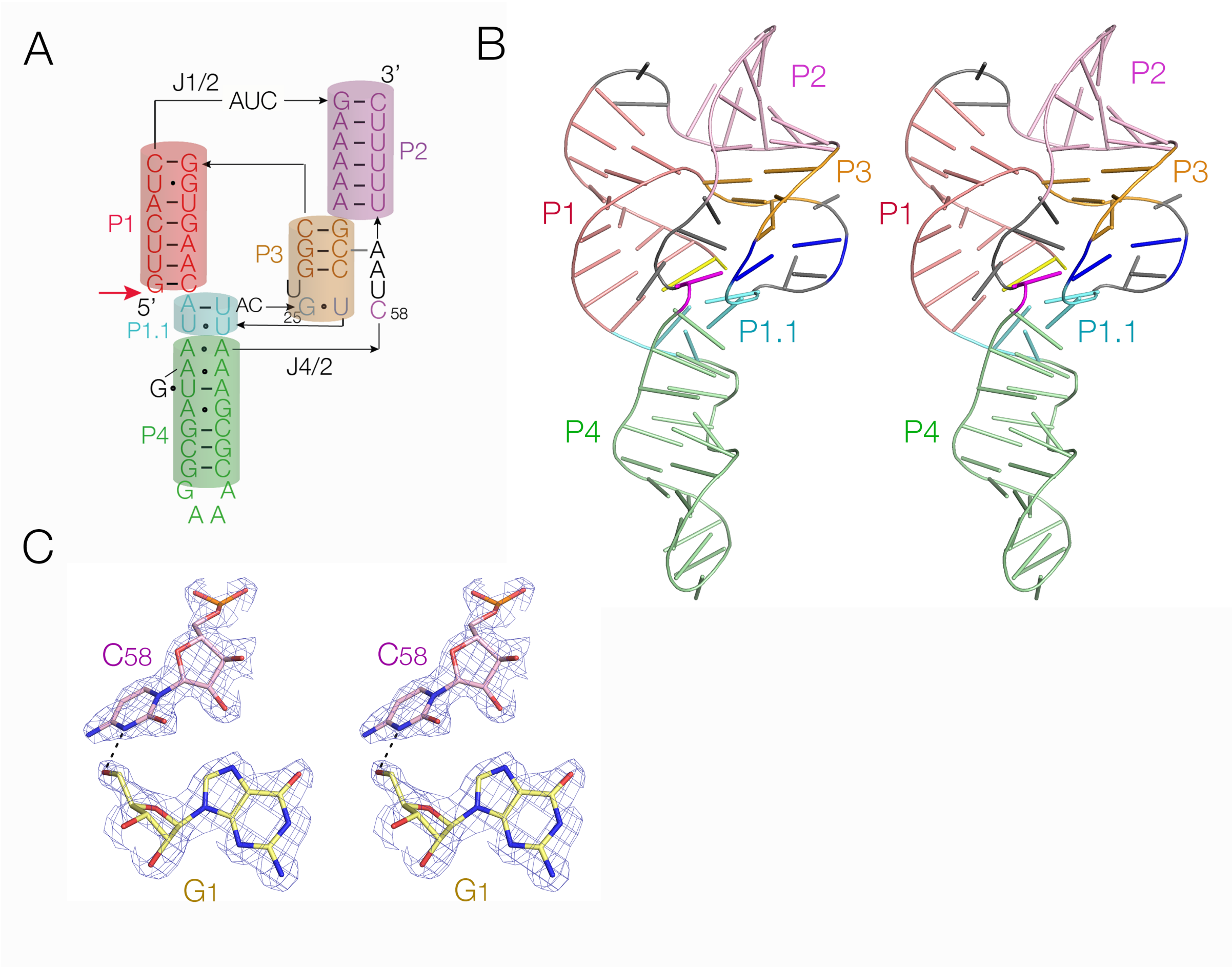

Supplementary Figure S5. The crystal structure of *Ackermannviridae* HDV ribozyme post-cleavage drawn as a parallel-eye stereoscopic images. **A**. Scheme of structure. **B**. The structure of the complete ribozyme depicted in cartoon mode. The 5'-terminal G1 is colored yellow, C58 is colored magenta and the G20:U25 basepair is colored blue. **C**. the active center of the ribozyme showing the electron density map contoured at 1.5 σ. Like the post-cleavage structure of the *C. briggsae* HDV ribozyme, the C58 N3 is adjacent to the O5' atom. Parallel-eye stereoscopic images are shown.

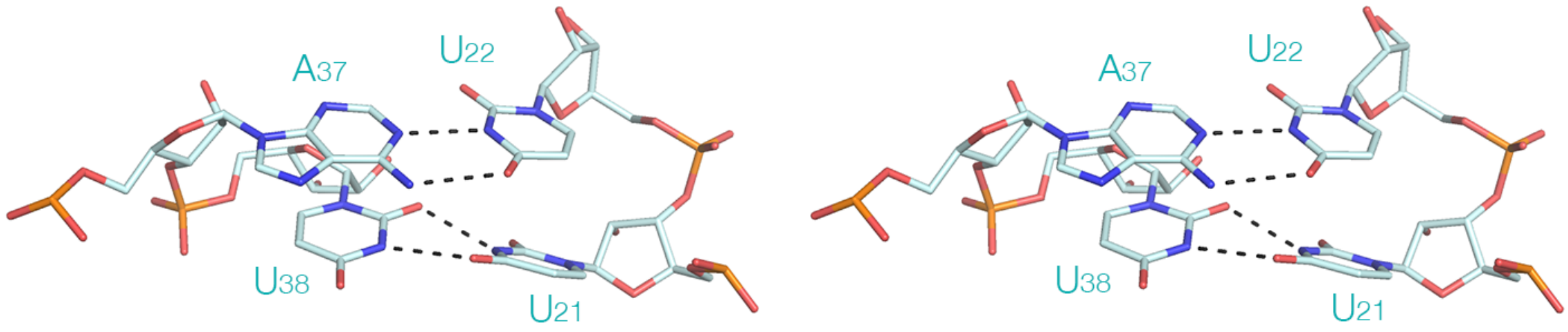

Supplementary Figure S6. The structure of the P1.1 helix of the *Ackermannviridae* HDV ribozyme post-cleavage. The helix comprises U:A and a U:U base pairs. Both are cis Watson-Crick base pairs connected by two hydrogen bonds. A parallel-eye stereoscopic image is shown.

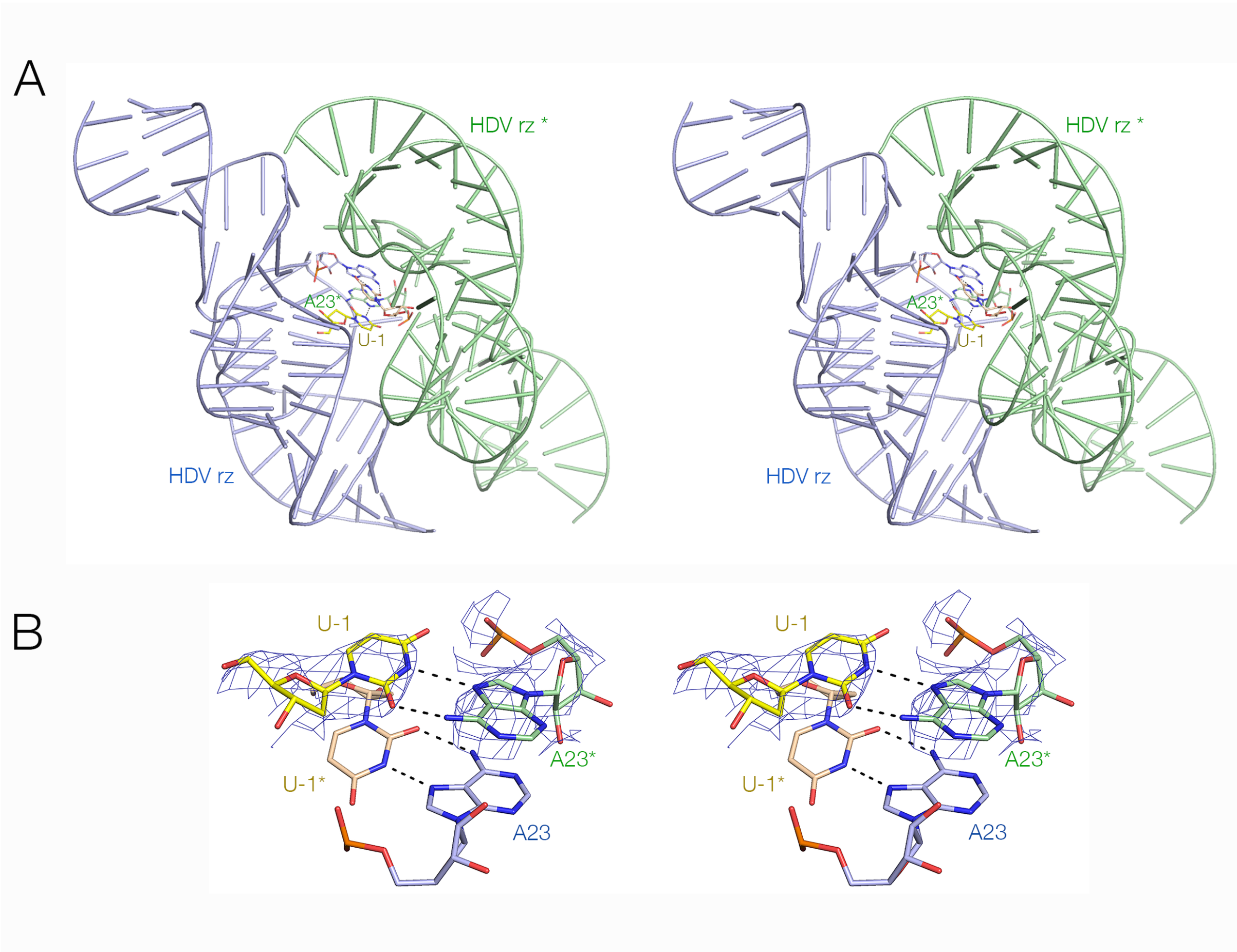

Supplementary Figure S7. Dimerization of the *C.briggsae* HDV ribozyme in the crystal lattice. **A**. Close interaction of two ribozyme molecules colored blue and green. **B**. Close-up view of the mutual base pairing between C-1 of one ribozyme with A23 of the other, with nucleobase base stacking between the two cytosines. The electron density contoured at 1.5 σ is shown for one of the base pairs. Parallel-eye stereoscopic images are shown.

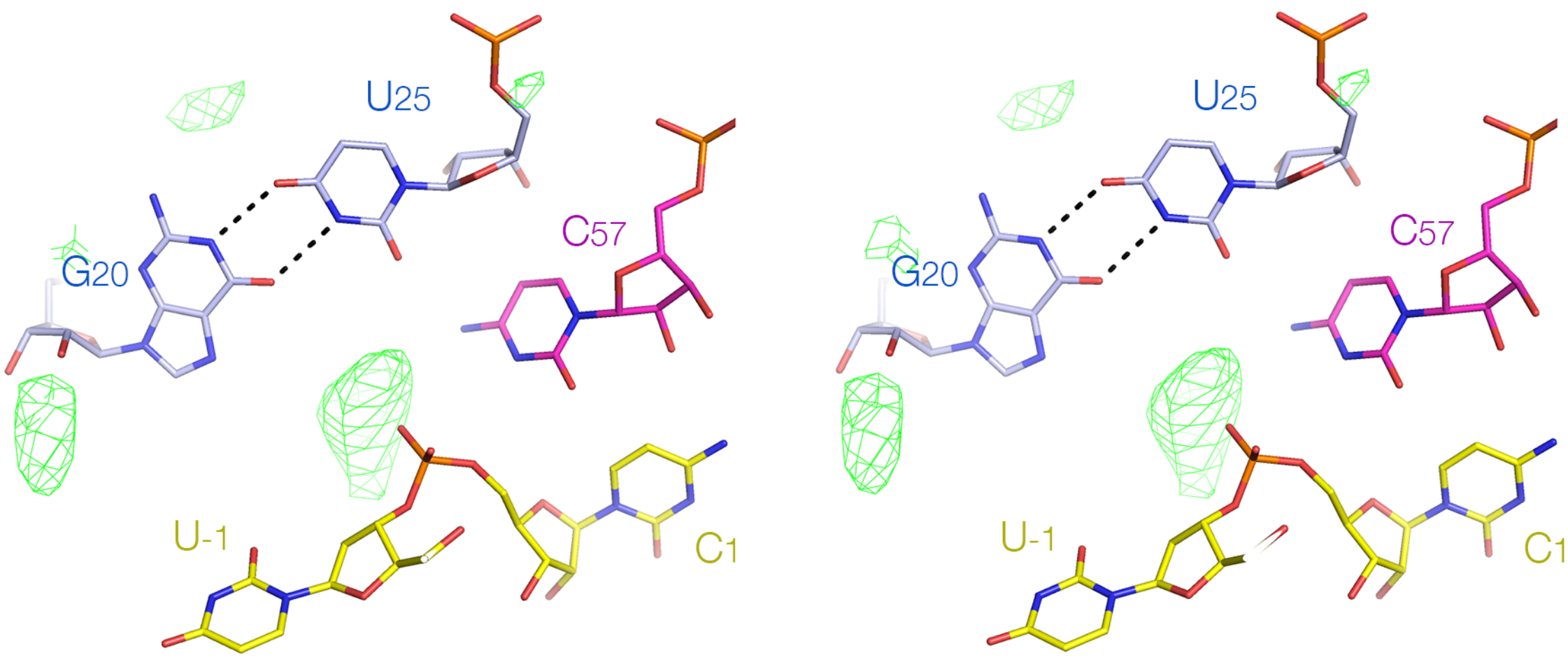

Supplementary Figure S8. A probable Mg^{2+} binding site adjacent to the scissile phosphate in the *C.briggsae* HDV ribozyme pre-cleavage structure. An **Fo-Fc** map contoured at 3σ (green) shows unassigned electron density.

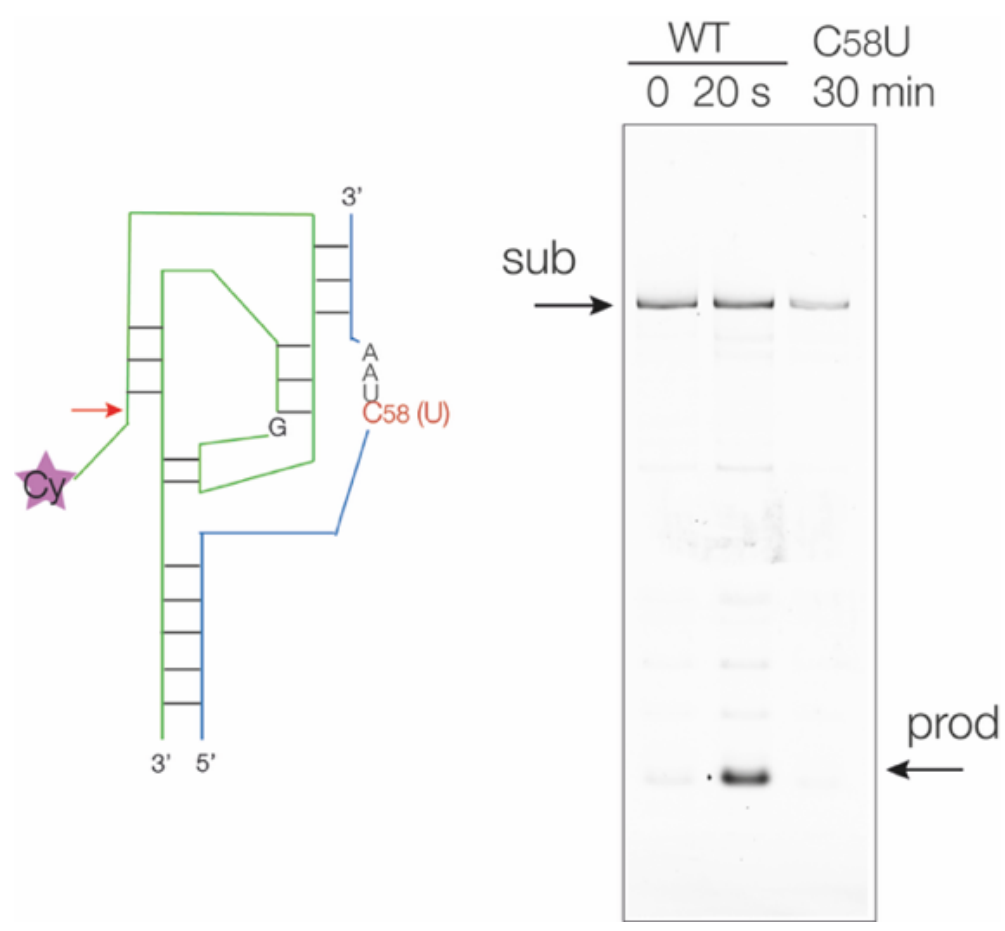

Supplementary Figure S9. Effect of a C58U mutation on the catalytic activity of the *Ackermannviridae* HDV ribozyme. The ribozyme was divided at the loop of the P4 helix (as shown on the left) and fluorescently-labeled with Cy3 at the 5' terminus. Incubation was performed under standard conditions containing 2 mM Mg^{2+} ions at 37°C for the times shown. Note that no detectable substrate cleavage has occurred during 30 min incubation. NB in the schematic (left) the bars indicate base pairing, but not the exact number of base pairs.

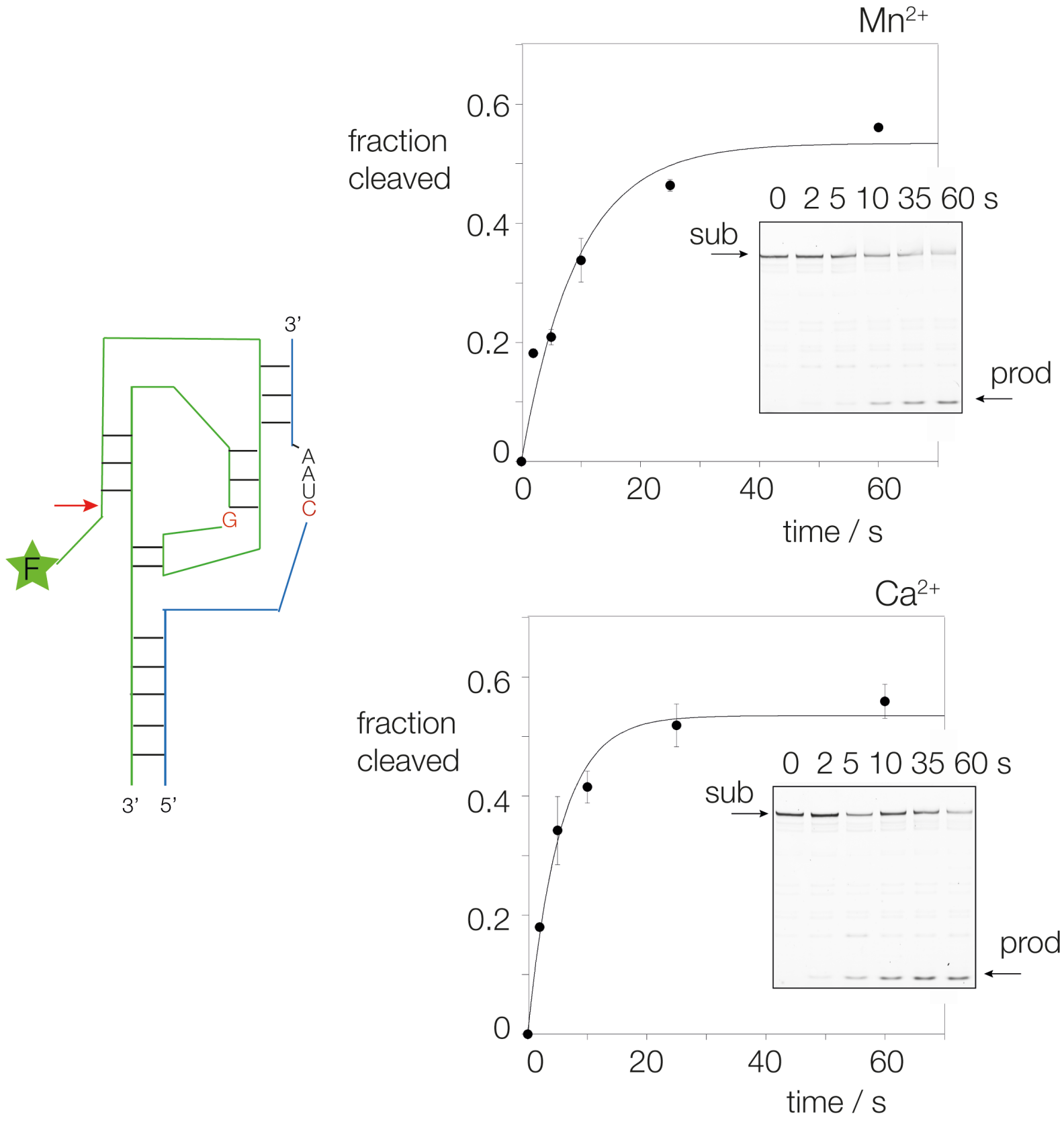

Supplementary Figure S10. Reaction progress of the *C.briggsae* HDV ribozyme under standard conditions containing 2 mM Mn^{2+} or Ca^{2+} ions at 37°C. The data have been fitted to single exponential functions giving observed cleavage rates characterized by $k_{obs} = 6.7 \pm 1.59$ and 11.1 ± 1.45 min^{-1} for Mn^{2+} (upper data) or Ca^{2+} (lower data) ions respectively (Table 1).

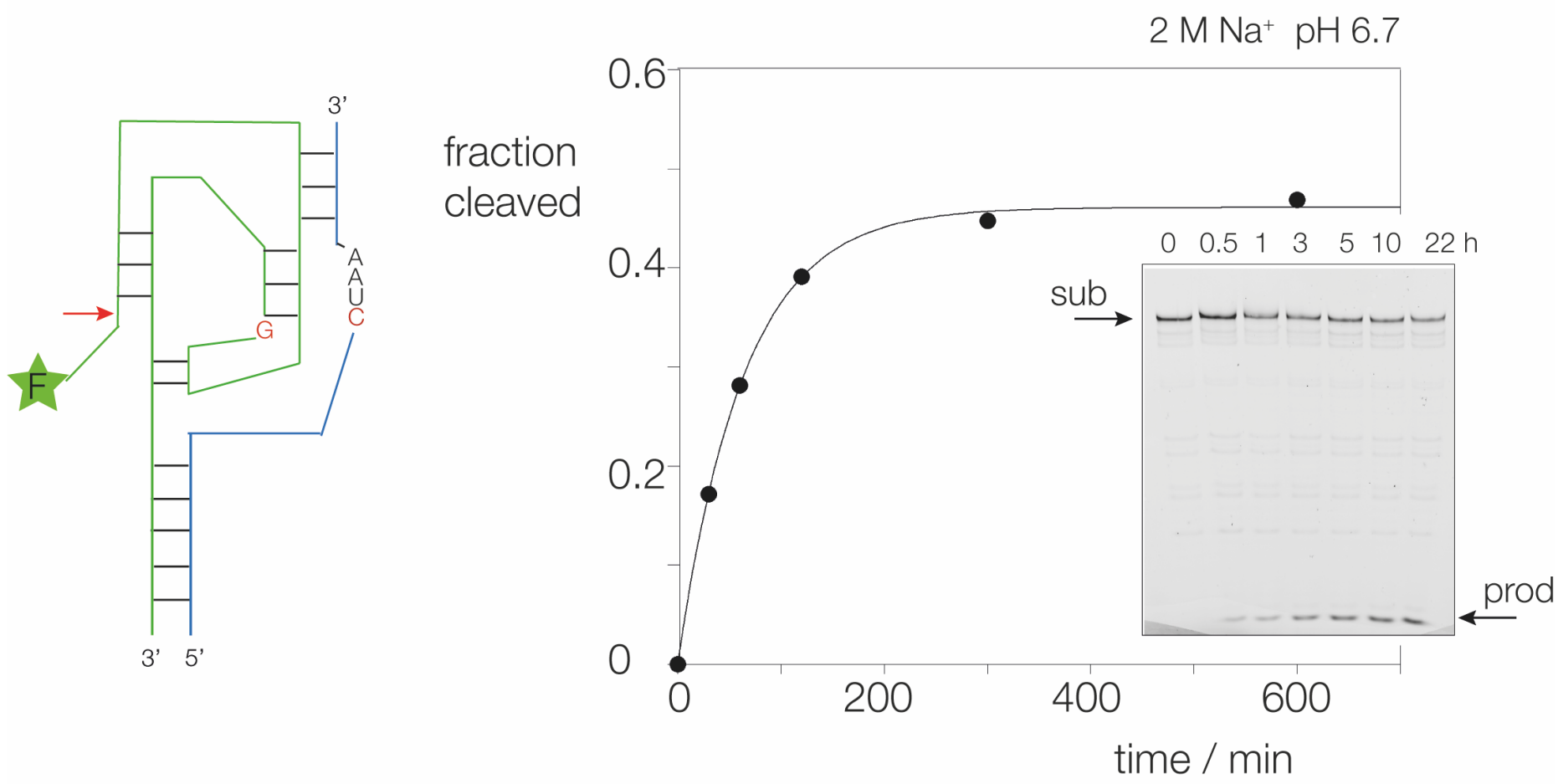

Supplementary Figure S11. Reaction progress of the *C.briggsae* HDV ribozyme in 20 mM MES (pH 6.7) containing 2 M Na^{+} ions at 37°C. The data have been fitted to a single exponential function giving an observed cleavage rate characterized by $k_{obs} = 0.01$ min^{-1}.

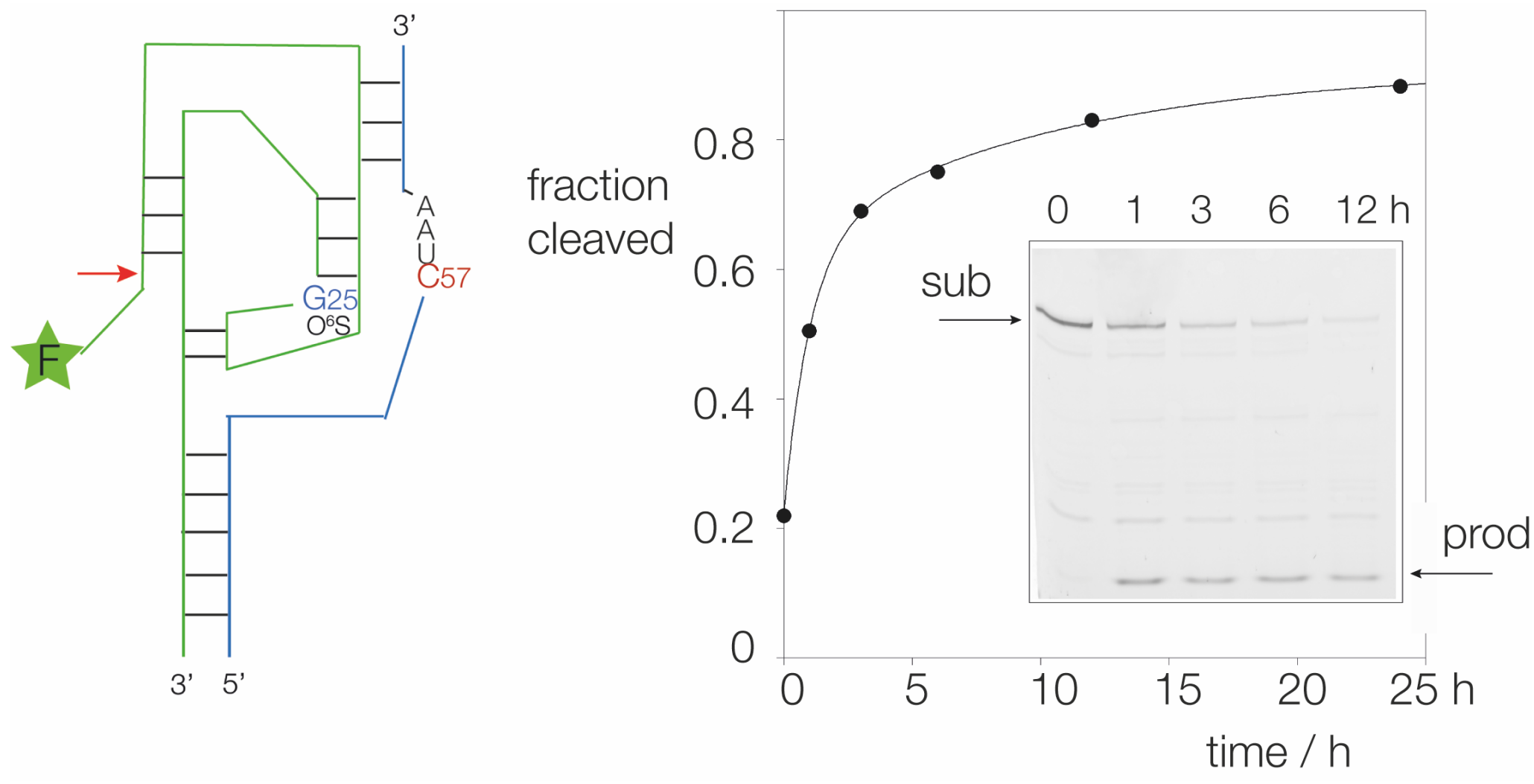

Supplementary Figure S12. Reaction progress for cleavage by the *C. briggsae* HDV ribozyme carrying a G25 O^6S substitution under standard conditions containing 2 mM Mg^{2+} ions at 37°C. The ribozyme was divided at the loop of the P4 helix (as shown on the left) and fluorescently-labeled with fluorescein at the 5' terminus. The data for this long incubation were fitted to two exponential functions, suggesting that the active conformation of the ribozyme is in slow exchange with an inactive form. The faster rate is characterized by $k_{obs} = 0.01\ h^{-1}$.

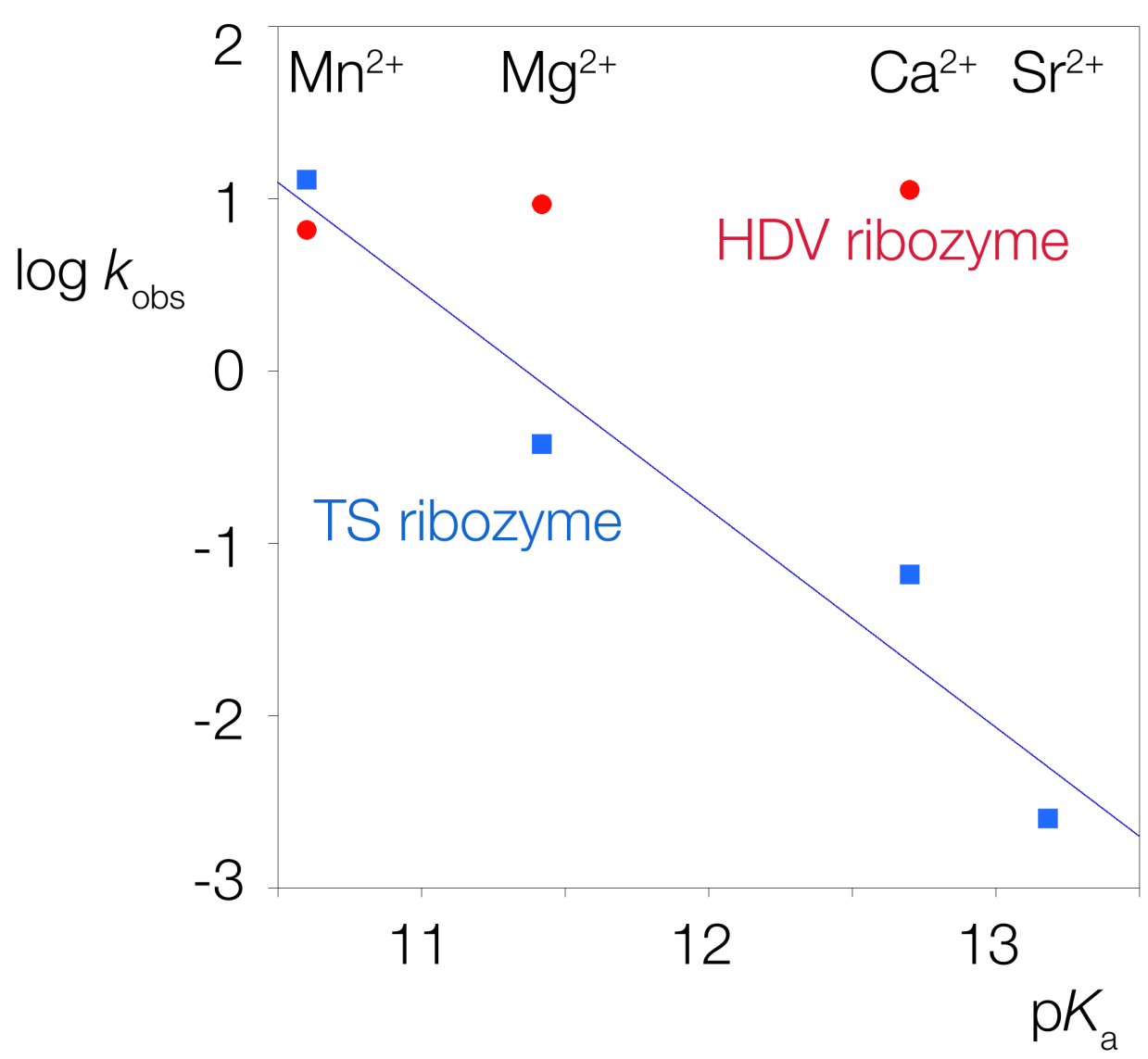

Supplementary Figure S13. Comparison of the dependence of observed cleavage rate vs metal ion pK_a for the *C. briggsae* HDV ribozyme (red) and the TS ribozyme (blue) where the metal ion acts as general base. The data for the TS ribozyme are taken from our earlier study [Y. Liu, T. J. Wilson and D. M. J. Lilley *Nature Chem. Biol* **13**, 508–513 (2017)], and fitted to a log-linear dependence (blue line).

SUPPLEMENTARY TABLES

Supplementary Table S1

J1/2 division

Ackermannviridae

GCAACCAGUUCAUC

GAAAAAGCCUUUACGUGGCGGUGAACAUAAGUAGCGGAAACGCGAAACUAAUUUUUC

C. briggsae

CCAUUUUCCCUUUC

GUACUGAGCCUCUACGUGGCGAAAGGGAUCAACAGACGAAAGUCUGGCUAAUCAGUAC

P4 division

Ackermannviridae

Cy3-GCAUCCAGUUCAUCAUCGAAAAAGCCUUUACGUGGCGGUGAACAUAAGUAGCGUCG

CGACGCGAAACUAAUUUUUC

C. briggsae

Flu-CCAUUUUCCCUUUCAUGUACUGAGCCUCUACGUGGCGAAAGGGAUCAACAGACUCGU

ACGAGUCUGGCUAAUCAGUAC

Supplementary Table S1 Sequences of RNA molecules used in the analysis of ribozyme cleavage and kinetics. All sequences written 5' to 3'. In studying ribozyme cleavage using the J1/2 division, the short 5' strands were radioactively [5'-^{32}P] labelled. Using the P4 division the long 5' strands were labelled with fluorophores at the 5' termini using Cy3 (*Ackermannviridae*) or fluorescein (Flu, *C. briggsae*).

Supplementary Table S2

Name	Sequence（5' seq 3'）	Notes
T7+6nt leader+ *C. briggsae* HDV DNA template	TAATACGACTCACTATAGCGTCGCCCCTTTCATGTACTGAGCCTCTACGTGGCG AAAGGGATCAACAGACGAAAGTCTGGCTAATCAGTAC	T7 promoter + 6-nt leader + HDV ribozyme
Full-length RNA transcript	GCGUCGCCCUUUCAUGUACUGAGCCUCUACGUGGCGAAAGGGAUCAACAGA CGAAAGUCUGGCUAAUCAGUAC	before self-cleavage
C. briggsae HDV ribozyme RNA	CCCUUUCAUGUACUGAGCCUCUACGUGGCGAAAGGGAUCAACAGACGAAAG UCUGGCUAAUCAGUAC	self-cleaved product
Pre-cleaved *C. briggsae HDV* ribozyme RNA (chemically synthesized)	NC01_dU_P4_7:（dU)CCCUUUCAUGUACUGAGCCUCUACGUGGCGAAAGGGAUC AACAGACG<br>NC01_P4-8：UCGUCUGGCUAAUCAGUAC	pre-cleaved
T7+7nt leader+ *Ackermannviridae* HDV DNA template	TAATACGACTCACTATAGCGTCGCGTTCATCATCGAAAAAGCCTTTACGTGGC GGTGAACATAAGTAGCGGAAACGCGAAACTAATTTTTC	T7 promoter + 7-nt leader + HDV ribozyme
Full-length RNA transcript	GCGUCGCGUUCAUCAUCGAAAAAGCCUUUACGUGGCGGUGAACAUAAGUAG CGGAAACGCGAAACUAAUUUUUC	before self-cleavage
Ackermannviridae HDV ribozyme RNA	GUUCAUCAUCGAAAAAGCCUUUACGUGGCGGUGAACAUAAGUAGCGGAAAC GCGAAACUAAUUUUUC	self-cleaved product

Supplementary Table S2. Nucleic acid sequences used in the crystallographic study of the *Ackermannviridae* and *C. briggsae* HDV-like ribozymes.

Supplementary Table S3

PDB	Crystallization conditions	cryo	nts	res	Space group	Beamline	Wavelength	Phasing
**HDV precleavage *Cb* NC01**	0.2 M magnesium chloride hexahydrate, 0.1M HEPES sodium (pH 7.5), 30% v/v polyethylene glycol 400	direct	48+ 19	2.95	P 6_5 2 2	BL10U2	0.9792	MR (22KS)
	NC01_dU_P4_7：(dU)CCCUUUCAUGUACUGAGCCUCUACGUGGCGAAAGGGAUCAACAGACG<br>NC01_P4-8：UCGUCUGGCUAAUCAGUAC							
**HDV cleaved *Cb* NC01**	0.2 M ammonium chloride, 0.01 M calcium chloride 0.05 M Tris.HCl (pH 8.5) 28% w/v polyethylene glycol 4000	direct	68	2.23	C 1 2 1	BL02U1	0.9786	MR (1DRZ)
	CCCUUUCAUGUACUGAGCCUCUACGUGGCGAAAGGGAUCAACAGACGAAAGUCUGGCUAAUCAGUAC							
**HDV cleaved *Ackermannviridae***	0.04 M lithium chloride, 0.08 M strontium chloride hexahydrate, 0.02 M magnesium chloride hexahydrate 0.04 M sodium cacodylate trihydrate (pH 7.0) 30% v/v (+/-)-2-methyl-2,4-pentanediol 0.012 M spermine tetrahydrochloride	direct	67	1.90	P 2_1 2_1 2	BL10U2	0.9792	MR (22KS)
	GUUCAUCAUCGAAAAAGCCUUUACGUGGCGGUGAACAUAAGUAGCGGAAACGCGAAACUAAUUUUUC							

Supplementary Table S3. Sequences and conditions employed in crystallization trials

Cb : C. briggsae ; *cryo*: cryoprotectant; *res*: resolution ; MR: molecular replacement using the structure in parenthesis.

Supplementary Table S4

Name	HDV pre-cleavage *C.briggsae* NC01	HDV post-cleavage *C.briggsae* NC01	HDV post-cleavage *Ackermannviridae*
PDB	22KX	22KS	22KW
**Data collection**			
Space group	P 6_5 2 2	C 1 2 1	P 2_1 2_1 2
Cell dimensions			
a, *b*, *c* (Å)	85.55 85.55 281.18	188.70 47.43 49.91	93.24 61.14 62.16
α, β, γ (°)	90.0 90.0 120.0	90.0 96.9 90.0	90.0 90.0 90.0
Phasing (PDB)	MR (22KS)	MR (1DRZ)	MR (22KS)
Wavelength	0.9792	0.9786	0.9786
Resolution (Å)	140.59 - 2.95 (3.11 - 2.95)	49.55 - 2.23 (2.35 - 2.23)	51.72 - 1.90 (2.01 - 1.90)
R_{merge}	0.075 (1.766)	0.118 (3.232)	0.059 (0.583)
R_{pim}	0.018 (0.429)	0.055 (1.589)	0.028 (0.366)
$I / \sigma I$	20.1 (1.9)	7.1 (0.9)	18.1 (2.9)
CC (1/2)	1.000 (0.820)	0.995 (0.327)	0.998 (0.799)
Completeness (%)	92.6 (99.9)	95.4 (89.8)	98.9 (93.4)
Redundancy	18.2 (17.7)	5.8 (5.4)	9.1 (5.7)
**Refinement**			
Resolution (Å)	26.83 - 2.95 (3.06 - 2.95)	31.22 - 2.24 (2.32 - 2.24)	26.32 - 1.90 (1.97 - 1.90)
No. reflections	12598 (1320)	19943 (1489)	28224 (2513)
R_{work} / R_{free}	0.197 / 0.244	0.221 / 0.247	0.201 / 0.241
Number of non-hydrogen atoms	2832	2912	3077
macromolecules	2826	2846	2852
ligands	6	4	20
water	0	62	205
Average *B*-factors	130.19	77.89	50.36
ligands	118.65	69.84	39.18
water		51.71	44.69
R.m.s. deviations			
Bond lengths (Å)	0.003	0.004	0.014
Bond angles (°)	0.90	0.93	1.95

*Values in parentheses are for highest-resolution shell.

Supplementary Table S4. Details of data collection and refinement statistics for the crystallographic data as deposited with the PDB.
