# Supplementary material for: Structure and mechanism of HDV ribozymes revealing Lewis acid catalysis by a metal ion
Source: Nat Commun. 2026 Sep 2;17:10462. doi: 10.1038/s41467-026-77452-w (PMC13635239; doi:10.1038/s41467-026-77452-w)
Supplement: Supplementary file 3 [file 41467_2026_77452_MOESM3_ESM.pdf]

nature portfolio

Peer Review File

Structure and mechanism of HDV ribozymes revealing Lewis acid catalysis by a metal ion

Corresponding Author: Professor David Lilley

This file contains all reviewer reports in order by version, followed by all author rebuttals in order by version.

Version 0:

Reviewer comments:

Reviewer #1

(Remarks to the Author)
This manuscript by Luo et al. and Lilley describes structural and kinetic analyses of two representatives of the long-studied HDV self-cleaving ribozyme class. Deep understanding of how RNAs catalyze chemical reactions has been hard won, and the present study helps to reveal how self-cleaving ribozymes accelerate RNA cleavage independent of protein enzymes. The research is generally well conducted and I have no concerns about the major conclusions. The authors should consider one major comment and a few minor comments listed below.

Major Comment
1. The authors conduct kinetic analyses of ribozyme function at different pH values and then make conclusions regarding mechanisms and the functional groups involved in catalysis. However, the authors do not state the temperature at which the Tris buffers were prepared. Tris is among the worst buffers for changing its pKa with changing temperature (delta pKa = -0.031 per degree C). If the buffers were prepared at room temperature (~22C) and the assays were conducted at 37C, then the pH curves are incurred and the proposed pKa values are off by ~0.3 to 0.45 units. The authors should consider this issue and make appropriate modifications to the plots and the conclusions if needed.

Minor Comments
2. In paragraph 2 of the Introduction, the organization of ribozymes into two categories seems imperfect. Some self-cleaving ribozymes are metalloenzymes that appear to use a divalent metal at the active site, including the RNAs under study in the current manuscript. How do these RNAs differ from the other metalloenzymes such that they need to be put into a separate category? The paragraph really could be deleted without any loss to the reader.
3. Lines 153 and 154, the speed of the rate of the ribozyme does not necessarily prove that the chemical step is rate limiting. The authors should maintain caution when discussing whether the observed pH profiles are due to a functional group directly involved in catalysis or one that might permit the active structure to fold correctly.
4. Line 236, the argument about why the higher pKa cannot be measured seems faulty. Data from testing ribozyme function at pH values around 10 or higher cannot be trusted because the folded RNA structure is likely to be disrupted (e.g. passing through the pKa values of functional groups forming base-pairs, e.g. pKa of G is ~9.5), rather that the “lability” (or chemical stability) of RNA due to internal phosphoester transfer involving the 2’ hydroxyl group (pKa is ~13.5).
5. What is the nature of the extra (very faint) band in the gels in Fig. 5?
6. In Fig. 6, I do not see why the N of C57 is marked as positively charged.

Reviewer #2

(Remarks to the Author)
The manuscript by Luo et al describes a structural and biochemical analysis of a eukaryotic and viral hepatitis delta virus (HDV) ribozyme. Recently, bioinformatic work by several groups have revealed that this class of ribozyme is broadly distributed across life, including within humans (CPEB3). The HDV ribozyme has served as a model system for understanding the structure and mechanism of small nucleolytic RNA enzymes, including extensive structural and mechanistic analysis (including groundbreaking work by the Lilley group) to determine the most likely mechanism by which this RNA catalyzes backbone scission. In the current work, the authors seek to extend prior analysis of several model HDV

ribozymes to a eukaryotic ribozyme from a nematode (C. biggsae) and a viral (Ackermannviridae). In this work, important findings focus on the crystal structure of the nematode ribozyme in both the pre- and post-cleavage states and associated biochemical analysis of both ribozymes to determine whether they share a common mechanism with other HDV ribozymes. These data clearly demonstrate that these ribozymes share a common structure (both globally and locally around the catalytic pocket) and a common mechanism. The conclusions of this work build upon the extensive database of knowledge of the ribozyme, with clear implications for the commonality of these RNAs across the entire family.

Overall, this work is both technically well-executed and the manuscript is clearly written, such that the primary conclusions are easy to interpret and understand. There is little that the authors can do to improve this work and thus, with very minor corrections, should be suitable for publication.

Minor comments:

Line 171—Why is it “curious” that there is only a single multiple base interaction in the ribozyme? (While the reviewer also finds this a interesting feature, other readers may require an additional sentence of context basically stating that it is unusual for an RNA aptamer or ribozyme to be reliant almost entirely on base pair interactions rather than more complicated triple and quadruple pairings).

Line 175—The authors sold double check their use of nomenclature for atoms such that it is consistent throughout the work. In line 170 they use a space between base and atom, whereas in 175 (and lines 193) there is no space. The former is better or the use of parenthesis like U(N3).

Line 203. I fully understand what the authors are saying here, but it might be more clear to use the term “lattice contact” in this sentence so that it is unambiguous as to why U(-1) shows up with clearly defined density.

Line 313. Spell out “approximately” instead of a ~ symbol.

Line 348. Spell out “twisted sister” at first use of TS ribozyme.

Reviewer #3

(Remarks to the Author)
In this study from the Huang and Lilley groups, the mechanisms of two newly discovered HDV ribozymes are investigated. These ribozymes hail from C. briggsae and the virus Ackermannviridae. The authors beautifully solve new crystal structures (one in the pre-cleaved and two in the product form) and confirm that the structures are akin to the original one from hepatitis delta virus, and that the role of C75/57 as the general acid is conserved. The novel claim of this study is that the metal ion in the reaction is a Lewis Acid, rather than a general base, as originally proposed by Bevilacqua and co-workers. Overall, this is a solid study that has strong structural and mechanistic data. I believe it deserved to be published in Nature Communications. However, there are alternative interpretations of the data that I would like the authors to consider including one in which the metal is both a Lewis acid and general base.

Major Comments
1. Nakano and co-workers showed that Cobalt-hexamine can act as a competitive inhibitor of the Mg2+. Since this is an outer-sphere species, it suggests that there is not the direct coordination of the 2’O needed for Lewis Acid catalysis. Have the authors tried cobalt hexamine inhibition experiments on their ribozymes?
2. It isn’t really clear why these two HDV ribozymes were studied. More justification for their choice is needed. Biological justification, if any, should be provided.
3. Throughout the manuscript, the authors call kobs a “rate”, however, it is a rate constant. A rate has units of M/s and a first order rate constant has units of s-1. This is more than semantics as rates and rate constants give different values in a A->B, A->C scenario. Please refer to them as rate constants.
4. Line 153. The notion that “very fast rate” means “chemistry is likely to be rate limiting” is incorrect. In fact, experiments where heavy atom isotope effects on ribozymes are measured and chemistry must be rate-limiting require a set up where the experiment goes as slow as possible. The authors can think of it like this: the slower chemistry is, the higher its peak is on the free energy profile and therefore the more likely it is to be rate-limiting. This sentence should be removed.
5. The P1.1 for Ackermannviridae in Fig S4 should show the detailed molecular interactions like in Fig 3C. Moreover, the P1.1s between Ackermannviridae and briggsae should be compared by RMSD. The P1.1 in these two structures is one of the most novel aspects to these structures in that here they are weak with AU and then a UU or UC; in contrast, they are GC then GC in the HDV ribozyme. I’d like to see more emphasis on this part of the paper, and perhaps an entire section as it makes the paper novel.
6. The density for the putative metal ion, shown in Fig 3D, appears to be modest, at least from this simple (and truncated) figure. The authors should provide an Fo-Fc map with the metal modeled and an Fo-Fc map without it modeled. They should also show an Fo-Fc with the metal ion modeled further than 2.1 Å away, like 3.5 Å away; and this plot should include more of the map than shown in Fig 3D. Together, these three Fo-Fc maps will give us an idea if the metal is needed and if it is better IS or OS.
7. Fig 4A. Point 1: The red curve should come down near pH 6.6 and the blue curve should come down near pH 10; right now this is reversed. The consequence of these corrections will not change the shape of the curve in Fig 4B, but they will change the y-axis, where the fAxfB (the active species with functional forms of the general AND general base) will be close to 10^-4. This leads to the prediction of a very fast kchem of ~1000 s-1, as predicted first by Nakano and co-workers

(Science, 2000, Ref 32) and similar to RNase A.
8. Fig 4A. Point 2: We are offered a mechanism in Figure 6E, in which OH2 is the base deprotonating the 2'OH; indeed, on lines 239 and 245, it is implicated that if the general base is not a hydrated metal ion, it could be solvent water. However, this does not fit the rate-pH profile in Fig 4A at all. Indeed, if solvent water were the base, the rate pH profile would be inverted from that measured, with a low pH plateau and high pH decreasing arm. To get the observed rate-pH profile, the base needs to be OH- or it could be "due to deprotonation of the O2' itself" (line 341). However, both of these models have severe problems. The pKa of OH- is 15.7 while that of O2' is similarly high. This means the fAxfB will be 10^-8 or so (the 10^-4 from the above comment with another 10^-4 from the difference in pKa of OH- and Mg(H2O) , which requires a kchem of 10^7 s-1, which is unrealistic when comparing to any RNase. This makes me favor general base catalysis by the metal ion, as originally proposed by Nakano. I would like the authors to consider that their metal ion could be both a Lewis acid and a general base, which Bevilacqua and Golden's data support, and try to model it as such. The authors will reply that if this is the case then the rate-pH profile should be like that in Fig S10 for TS. However, metal ion-RNA interactions are extraordinarily complex—much more so than in proteins—owing to the myriad interactions metal ions can have with the backbone. Perhaps Mg and Ca speed the reaction back up due to distal interactions, such as published at C41 in HDV by Bevilacqua and co-workers.
9. Line 256. "C1" should be "N3".
10. The 2 M Na+ experiments need large amounts of EDTA to assure that no contaminating divalents are driving the reaction, as first established in the HDV ribozyme by Been and co-workers.
11. Line 372. When the authors write "although the O2'-P distance was good, at 3 Å" do they mean "was well determined, at 3 Å"?
12. Line 399. 4.7 and 5.3 Å seem too far to be outer-sphere ligands to the metal ion. What precedent is there for such long distances?

Minor Comments
1. Line 50. Delete the comma.
2. Line 68. I think "DNA species" should be "RNA species".
3. The title on the SI doesn't match that on the main text.
4. Line 143. The kobs values should be added to Fig2 and/or legend.
5. Line 146. The 3 bp extension should be captured in the schematic.
6. Line 146. "The 5'end was extended by seven nucleotides from the expected cleavage site, so that the total length of the 5'RNA was 56 nt." There is no way to see this other than trust the authors. The nucleotide numbering should be shown in Figure 2.
7. Why did the authors switch from 5'-32P to 5'-fluorophore between J1/2 open and P4 open and what if anything is the consequence?
8. Line 168. C57 is not in the major groove of P3, as it isn't part of P3. One might say it is NEAR the major groove of P3.
9. Line 663. Remove the slash.
10. Line 411 delete ", and"

Reviewed signed: Phil Bevilacqua
Date: June 4, 2026

Version 1:

Reviewer comments:

Reviewer #1

(Remarks to the Author)
The authors have addressed all my points and I support publication without reservation.

Reviewer #3

(Remarks to the Author)
Comments on other Reviewers
Reviewer 1
1.) Response to Reviewer #1, Point 6. A double bond is needed between N3 and C4. As drawn, C4 doesn't have an octet. This is particularly important as this is now the concluding figure of the paper.

Reviewer 2
1.) The dialogue on Line 171 is quite interesting. I encourage the authors to write a future perspective on ribozymes concerning this feature.

My Review
1.) I appreciate the authors conducting the new experiment to test for inhibition by cobalt hexamine. Given that they (effectively) find no inhibition (Ki>10 mM), then I agree that their mechanism, with direct coordination of the 2'O to the Mg2+ ion, is realistic. At the same time, it is important that they acknowledge that the outcome of this inhibition was different in the original HDV ribozyme (Ki of 45 uM in Science 2000, footnote 22), which suggests that the mechanistic role for the metal ion might differ between HDV ribozymes. I think this should be mentioned.

2.)3.) I agree with changes.

4.) I think this explanation is fine, but it is important to point out that these considerations are at neutral pH. We can always take the pH down to 4 and make chemistry the rls, as Harris, Piccirilli, and York have successfully done.

5.) The new additions strengthen the paper and are really appreciated. Thank you.

6.) The new Fo-Fc maps significantly strengthen the paper, and the authors' honest grappling with the limitations of the data are appreciated.

7.) This now looks good. Glad this was caught!

8.) I'm satisfied that the authors write "We agree that water acting as the base is not consistent with our observed pH dependence". And of course, it is not necessary to know everything to publish (or none of us would ever publish). I'm satisfied with this change. With that said, it is also possible that the proton transfer agent is buffer/metabolite, as we demonstrated under certain conditions for twister (Messina and Bevilacqua, 2018) and others have protein enzymes. The authors may want to entertain this possibility and indeed test it in a future study.

9.)-12.) I agree with changes.

Minor Revisions: I agree with changes.

Review signed: Phil Bevilacqua
Date: July 14, 2026

Open Access This Peer Review File is licensed under a Creative Commons Attribution 4.0 International License, which permits use, sharing, adaptation, distribution and reproduction in any medium or format, as long as you give appropriate credit to the original author(s) and the source, provide a link to the Creative Commons license, and indicate if changes were made.
In cases where reviewers are anonymous, credit should be given to 'Anonymous Referee' and the source.
The images or other third party material in this Peer Review File are included in the article's Creative Commons license, unless indicated otherwise in a credit line to the material. If material is not included in the article's Creative Commons license and your intended use is not permitted by statutory regulation or exceeds the permitted use, you will need to obtain permission directly from the copyright holder.
To view a copy of this license, visit https://creativecommons.org/licenses/by/4.0/

NCOMMS-26-028496-T Luo … Lilley Lewis acid catalysis by a metal ion in a nucleolytic ribozyme; structure and mechanism of new HDV ribozymes

We are very grateful to all three reviewers for their careful reading of our paper, and their helpful comments. These gave us lots of food for thought on the catalytic mechanism, and how to present our ideas clearly. As a result of this we have significantly revised our manuscript, fairly substantially in the case of the Discussion where we have also changed the ordering to make the logic clearer. We believe this is now a substantially improved paper.

With best regards David Lilley

Responses to comments from the reviewers.

Reviewer #1 (Remarks to the Author)

This manuscript by Luo et al. and Lilley describes structural and kinetic analyses of two representatives of the long-studied HDV self-cleaving ribozyme class. Deep understanding of how RNAs catalyze chemical reactions has been hard won, and the present study helps to reveal how self-cleaving ribozymes accelerate RNA cleavage independent of protein enzymes. The research is generally well conducted and I have no concerns about the major conclusions. The authors should consider one major comment and a few minor comments listed below.

Major Comment

1. The authors conduct kinetic analyses of ribozyme function at different pH values and then make conclusions regarding mechanisms and the functional groups involved in catalysis. However, the authors do not state the temperature at which the Tris buffers were prepared. Tris is among the worst buffers for changing its pKa with changing temperature (delta pKa = -0.031 per degree C). If the buffers were prepared at room temperature (~22C) and the assays were conducted at 37C, then the pH curves are incurred and the proposed pKa values are off by ~0.3 to 0.45 units. The authors should consider this issue and make appropriate modifications to the plots and the conclusions if needed.

The reviewer is quite correct, the pH of all the buffers (but Tris particularly) are temperature dependent. We recalculated the plot of rate vs pH with corrected pH values and have replaced Figure 4 in the paper. The shape of the curve remains closely similar, and the pK_a emerging from the fit changes from the original 6.6 to 6.5. The text has been corrected.

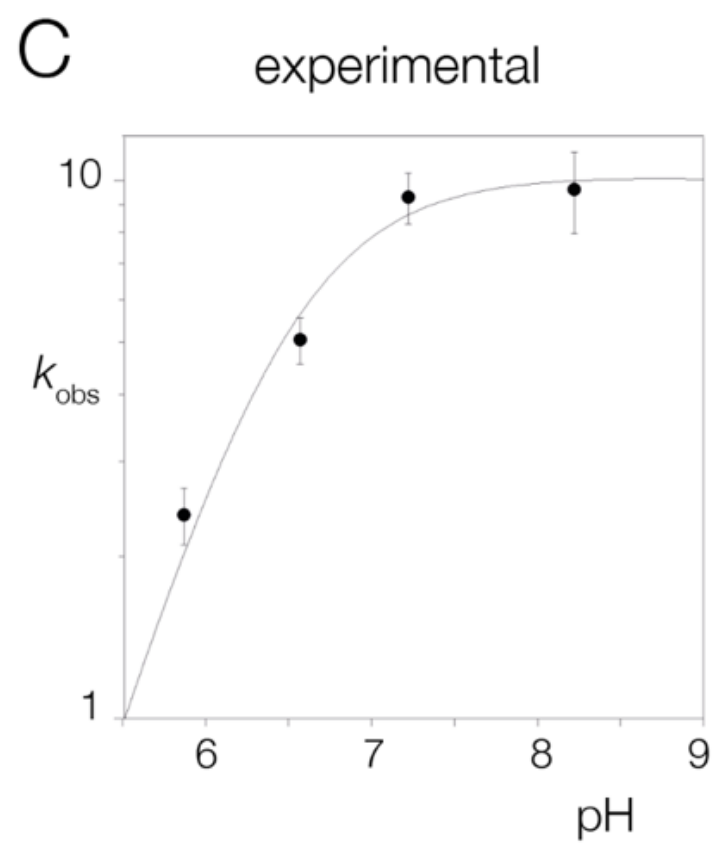

Minor Comments

2. In paragraph 2 of the Introduction, the organization of ribozymes into two categories seems imperfect. Some self-cleaving ribozymes are metalloenzymes that appear to use a divalent metal at the active site, including the RNAs under study in the current manuscript. How do these RNAs differ from the other metalloenzymes such that they need to be put into a separate category? The paragraph really could be deleted without any loss to the reader.

The real distinction between the two classes of ribozymes is mechanistic. In general the nucleolytic ribozymes use general acid-base catalysis (akin to RNaseA in the protein world). These ribozymes can exploit almost any functionality for this purpose, including nucleobases, 2'-OH and hydrated metal ions. The other class includes the intron ribozymes, these do not do use general acid-base catalysis, but rather use metal ions as Lewis acids to activate, and to organize the transition state. The distinction is not the use of metal ions, but rather a difference in catalytic mechanism. We have now changed the text to make this clearer. It is important to put this into the Introduction, as the conclusion of the paper suggests that HDV is now intermediate between the classes.

3. Lines 153 and 154, the speed of the rate of the ribozyme does not necessarily prove that the chemical step is rate limiting. The authors should maintain caution when discussing whether the observed pH profiles are due to a functional group directly involved in catalysis or one that might permit the active structure to fold correctly.

We agree that this is strictly correct. However a slow ribozyme is highly likely to be impeded by RNA folding. For example, the original simplified hammerhead ribozymes had rates of around k_{obs} = 0.05 min^{-1}. The extended hammerhead with additional tertiary contacts were faster at k_{obs} = 1 min^{-1}, and the recent pseudoknot-containing hammerhead is one of the fastest-known ribozymes at 16 min^{-1}. They all have the same active site. Clearly the slower ones are held back by slow RNA folding. But we do agree with the second sentence, and now discuss this point in the paper.

4. Line 236, the argument about why the higher pKa cannot be measured seems faulty. Data from testing ribozyme function at pH values around 10 or higher cannot be trusted because the folded RNA structure is likely to be disrupted (e.g. passing through the pKa values of functional groups

forming base-pairs, e.g. pKa of G is ~9.5), rather that the “lability” (or chemical stability) of RNA due to internal phosphoester transfer involving the 2’ hydroxyl group (pKa is ~13.5).

Probably all these effects become significant above pH = 9. So we do not measure rates in that high pH region and that is why it is greyed out in the figure.

5. What is the nature of the extra (very faint) band in the gels in Fig. 5?

We really don’t know the origin of these very faint cleavages. In general we observe a low level of cleavage in RNA that is somewhat sequence dependent.

6. In Fig. 6, I do not see why the N of C57 is marked as positively charged.
Clearly C57 must be protonated to act as general acid. We would expect the positive charge to be largely associated with the moving proton. We have therefore altered the figure in that regards.

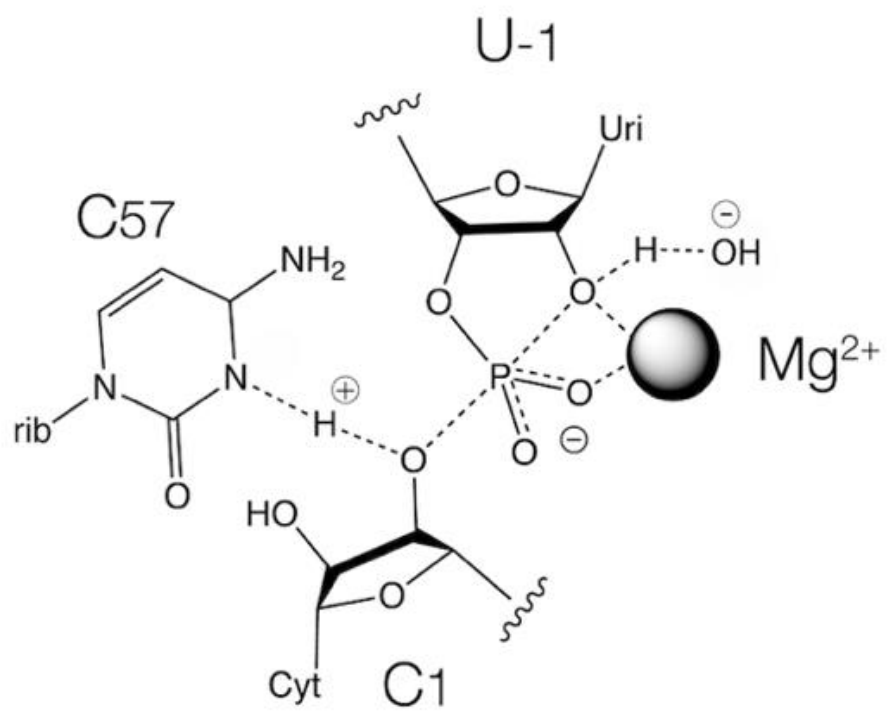

Reviewer #2 (Remarks to the Author)

The manuscript by Luo et al describes a structural and biochemical analysis of a eukaryotic and viral hepatitis delta virus (HDV) ribozyme. Recently, bioinformatic work by several groups have revealed that this class of ribozyme is broadly distributed across life, including within humans (CPEB3). The HDV ribozyme has served as a model system for understanding the structure and mechanism of small nucleolytic RNA enzymes, including extensive structural and mechanistic analysis (including groundbreaking work by the Lilley group) to determine the most likely mechanism by which this RNA catalyzes backbone scission. In the current work, the authors seek to extend prior analysis of several model HDV ribozymes to a eukaryotic ribozyme from a nematode (C. biggsae) and a viral (Ackermannviridae). In this work, important findings focus on the crystal structure of the nematode ribozyme in both the pre- and post-cleavage states and associated biochemical analysis of both ribozymes to determine whether they share a common mechanism with other HDV ribozymes. These data clearly demonstrate that these ribozymes share a common structure (both globally and locally around the catalytic pocket) and a common mechanism. The conclusions of this work build upon the extensive database of knowledge of the ribozyme, with clear implications for the commonality of these RNAs across the entire family.

Overall, this work is both technically well-executed and the manuscript is clearly written, such that the primary conclusions are easy to interpret and understand. There is little that the authors can do to improve this work and thus, with very minor corrections, should be suitable for publication.

Thank you for these kind comments.

Minor comments:

Line 171—Why is it “curious” that there is only a single multiple base interaction in the ribozyme? (While the reviewer also finds this a interesting feature, other readers may require an additional sentence of context basically stating that it is unusual for an RNA aptamer or ribozyme to be reliant almost entirely on base pair interactions rather than more complicated triple and quadruple pairings).

We have determined a lot of riboswitch and ribozyme structures and most have some multiple-base interactions. For example, the NADII riboswitch has seven triple and two quadruple-base interactions. It therefore struck us that there were so few in this highly-folded ribozyme structure. But really this is a subjective response based on what comparisons are in mind, so we have removed the adverb “curiously”.

Line 175—The authors sold double check their use of nomenclature for atoms such that it is consistent throughout the work. In line 170 they use a space between base and atom, whereas in 175 (and lines 193) there is no space. The former is better or the use of parenthesis like U(N3).

We have checked through and made this consistent e.g. “U38 N3”.

Line 203. I fully understand what the authors are saying here, but it might be more clear to use the term “lattice contact” in this sentence so that it is unambiguous as to why U(-1) shows up with clearly defined density.

We thought this was clear, but we have added “ As a result of this lattice contact, the measured ..” to clarify as requested.

Line 313. Spell out “approximately” instead of a ~ symbol.

This has been changed as requested.

Reviewer #3 (Remarks to the Author):

In this study from the Huang and Lilley groups, the mechanisms of two newly discovered HDV ribozymes are investigated. These ribozymes hail from C. briggsae and the virus *Ackermannviridae*. The authors beautifully solve new crystal structures (one in the pre-cleaved and two in the product form) and confirm that the structures are akin to the original one from hepatitis delta virus, and that the role of C75/57 as the general acid is conserved. The novel claim of this study is that the metal ion in the reaction is a Lewis Acid, rather than a general base, as originally proposed by Bevilacqua and co-workers. Overall, this is a solid study that has strong structural and mechanistic data. I believe it deserved to be published in *Nature Communications*. However, there are alternative interpretations

of the data that I would like the authors to consider including one in which the metal is both a Lewis acid and general base.

This review has been signed by Phil Bevilacqua. As always this is written with great insight into RNA catalysis and we are very grateful for these valuable points.

Major Comments

1. Nakano and co-workers showed that Cobalt-hexamine can act as a competitive inhibitor of the Mg^{2+}. Since this is an outer-sphere species, it suggests that there is not the direct coordination of the 2'O needed for Lewis Acid catalysis. Have the authors tried cobalt hexamine inhibition experiments on their ribozymes?

In response to this question we explored competition between the Mg^{2+} ion required for catalysis, and exchange inert cobalt(III) hexammine, using the very fast *C. briggsae* ribozyme (see the gel below). In the presence of our standard 2 mM Mg^{2+} ions inhibition of cleavage was only observed at 10 mM and above cobalt(III) hexammine. This suggests to us that the Mg^{2+} ion is bound tightly in the catalytic site such that it is not readily displaced by the triply-charged cobalt(III) hexammine. To our minds this is quite consistent with a Mg^{2+} ion bound as an inner-sphere complex for the *C. briggsae* HDV ribozyme.

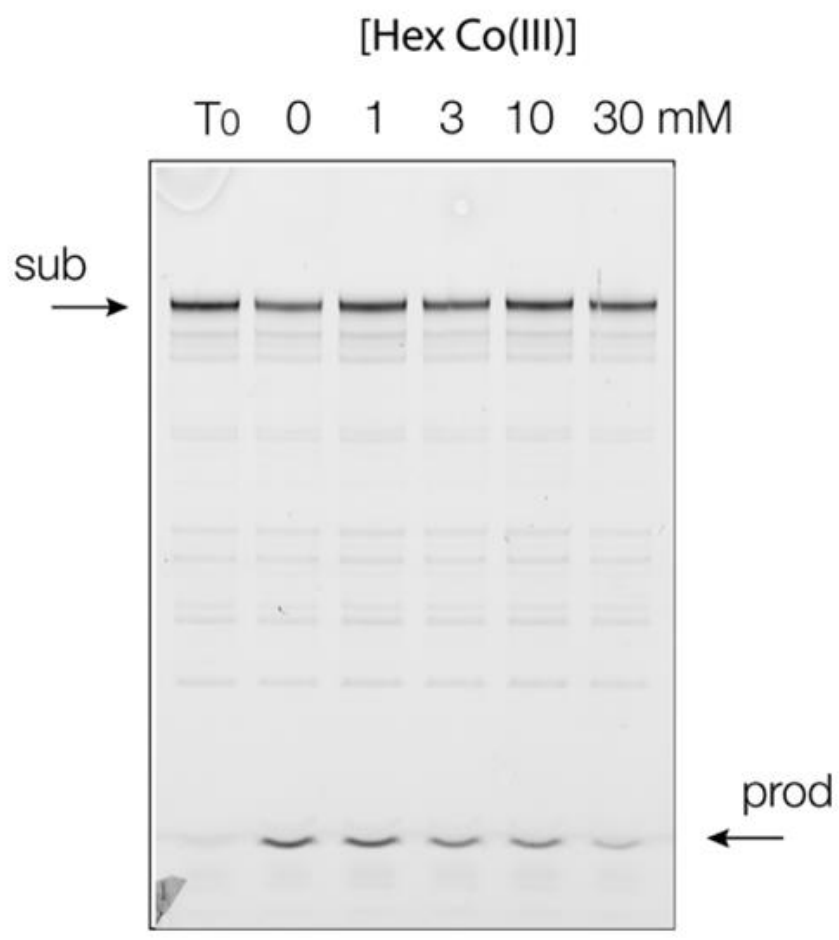

2. It isn't really clear why these two HDV ribozymes were studied. More justification for their choice is needed. Biological justification, if any, should be provided.

Our initial selection was guided by the sequence and structural diversity of the newly identified HDV ribozyme variants reported by Breaker and colleagues, with particular emphasis on differences in the P4 stem architecture. Specifically, the candidate set included ribozymes with distinct P4 stem features, including variants containing an E-loop motif and variants lacking this motif. Thus, these RNAs were chosen to sample naturally occurring structural diversity within the HDV-like ribozyme family, rather than to focus on a single biological source or organism. We also note that we experimentally screened multiple HDV-like ribozyme sequences for crystallization. Among the candidates tested, the two ribozymes reported here were the only ones that produced crystals diffracting to sufficiently high resolution for detailed structural analysis. Therefore, the final selection reflected both biological/structural diversity and crystallographic tractability. We have now clarified

the rationale for selecting these two HDV-like ribozymes in a separate first sub-section of the Results section.

3. Throughout the manuscript, the authors call k_{obs} a "rate", however, it is a rate constant. A rate has units of M/s and a first order rate constant has units of s^{-1}. This is more than semantics as rates and rate constants give different values in a A->B, A->C scenario. Please refer to them as rate constants.

When we say something like ".. with a rate of k_{obs} = X min^{-1}" what we really mean that the rate is characterised by a rate constant of X". We agree this is a bit sloppy - perhaps we tend to use rates and rate constants rather loosely. We have now tightened this up in the text.

4. Line 153. The notion that "very fast rate" means "chemistry is likely to be rate limiting" is incorrect. In fact, experiments where heavy atom isotope effects on ribozymes are measured and chemistry must be rate-limiting require a set up where the experiment goes as slow as possible. The authors can think of it like this: the slower chemistry is, the higher its peak is on the free energy profile and therefore the more likely it is to be rate-limiting. This sentence should be removed.

We have discussed this point above, see our response to Referee 1 point 3. The history of ribozyme study tends to show that what were initially slow ribozymes became much faster when elements that confer good folding (tertiary interactions in the hammerhead, the four-way junction in the hairpin etc) were included. When these are in place the rates tend to be in the region of ≥ 10 min^{-1}, where it seems likely that chemistry not folding is rate-limiting. Moreover, if we take a case where f_A x f_B is ~10^{-4} (e.g. pK_a values of say 5 and 9), then the corrected rate for active ribozyme molecules is around 10^5 min^{-1}, which is comparable with the protein enzyme RNaseA. We have clarified our discussion of this point in the section section of the Results (Division of the new HDV ribozymes …).

5. The P1.1 for *Ackermannviridae* in Fig S4 should show the detailed molecular interactions like in Fig 3C. Moreover, the P1.1s between *Ackermannviridae* and *briggsae* should be compared by RMSD. The P1.1 in these two structures is one of the most novel aspects to these structures in that here they are weak with AU and then a UU or UC; in contrast, they are GC then GC in the HDV ribozyme. I'd like to see more emphasis on this part of the paper, and perhaps an entire section as it makes the paper novel.

This is a good point, thank you. In the viral HDV ribozyme P1.1 comprises two C:G base pairs, whereas in our two structures it is a U:A and either U:C or U:U. The helix still comprises two co-planar base pairs, and each held by two hydrogen bonds (the *C. briggsae* P1.1 is shown in Figure 3C). But in principle, this should be a weaker mini-helix (with four, not six, hydrogen bonds in total) that may well explain why these two ribozymes are so much more sensitive to breaking the link between P1 and P2. As requested we have shown the corresponding P1.1 helix of the *Ackermannviridae* ribozyme in the revised SI (Supplementary Figure S5) as shown below, and expanded a discussion in the text. The structures of the P1.1 helices of *Ackermannviridae* vs *C. briggsae* post cleavage

superimpose with an RMSD = 0.371 Å. Those of the *C. briggsae* pre- and post-cleavage superimpose with an RMSD = 0.211 Å.

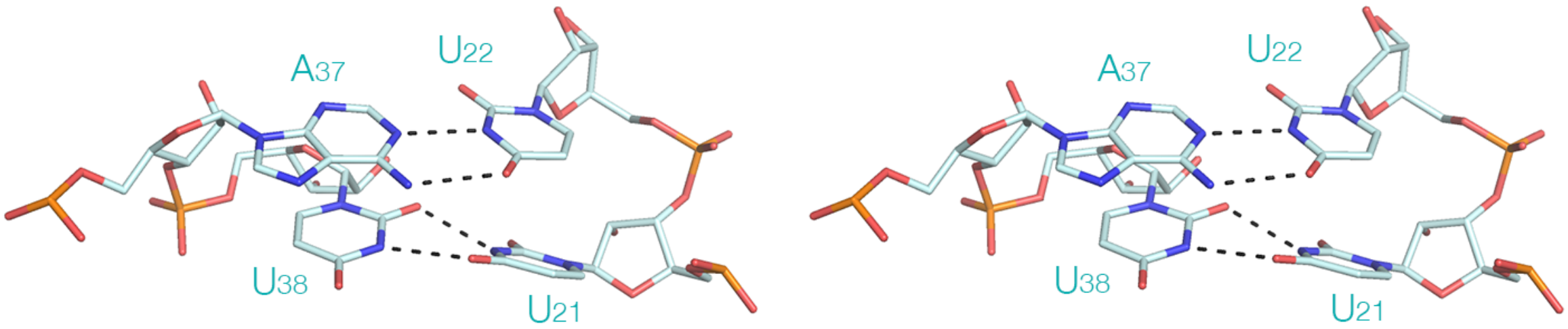

6. The density for the putative metal ion, shown in Fig 3D, appears to be modest, at least from this simple (and truncated) figure. The authors should provide an F_o-F_c map with the metal modeled and an Fo-Fc map without it modeled. They should also show an F_o-F_c with the metal ion modeled further than 2.1 Å away, like 3.5 Å away; and this plot should include more of the map than shown in Fig 3D. Together, these three F_o-F_c maps will give us an idea if the metal is needed and if it is better IS or OS.

We agree that the section of the 2**F**o-**F**c map shown in Figure 3 does not provide the best view of the density for the metal ion. We have calculated the **F**o-**F**c map for the active center, shown below. The **F**o-**F**c map at 3σ is colored green here. This shows very clearly that there is unassigned electron density around 2 Å from the *pro-R* non-bridging oxygen atom of the scissile phosphate. The distance is fully consistent with an inner-sphere interaction. However the resolution is not good enough to model the ion unambiguously with the precision being asked for. The models being requested can not be judged with the confidence sought. Nevertheless it is clear that the peak in the **F**o-**F**c map is much closer to the scissile phosphate than to the G:U base pair, consistent with direct bonding to the phosphate group. We agree that the **F**o-**F**c map should definitely be included with the data of the paper, and this stereo image has now been added to the SI as Supplementary Figure S7.

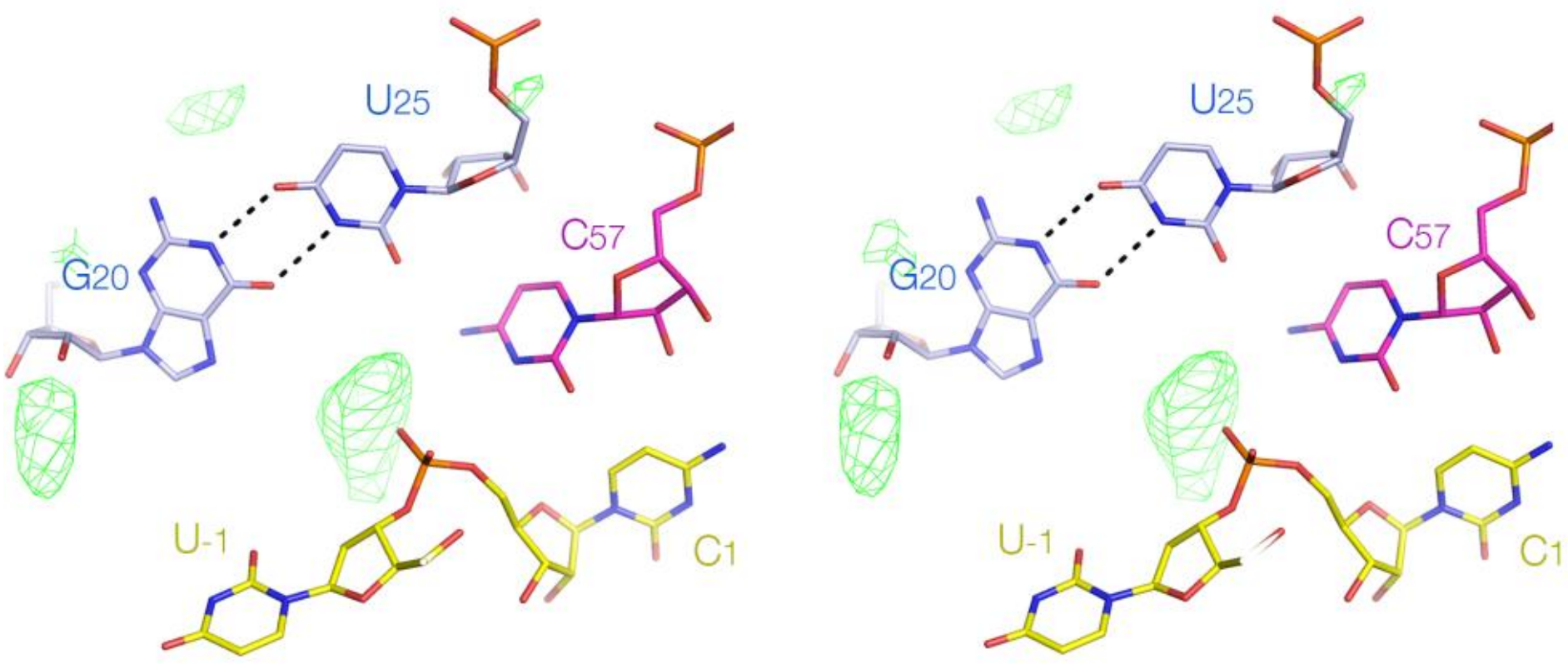

7. Fig 4A. Point 1: The red curve should come down near pH 6.6 and the blue curve should come down near pH 10; right now this is reversed. The consequence of these corrections will not change the shape of the curve in Fig 4B, but they will change the y-axis, where the $f_A x f_B$ (the active species with functional forms of the general AND general base) will be close to 10^-4. This leads to the prediction of a very fast k_{chem} of ~1000 s^{-1}, as predicted first by Nakano and co-workers (Science, 2000, Ref 32) and similar to RNase A.

Sorry, this was a total error on our part. Inversion of the pK_a values was done by mistake early on, the error was discovered and corrected. But somehow the wrong version was included with the submitted figures. This has now been corrected, and the correct plot shown (as reproduced below). Apologies for this confusion.

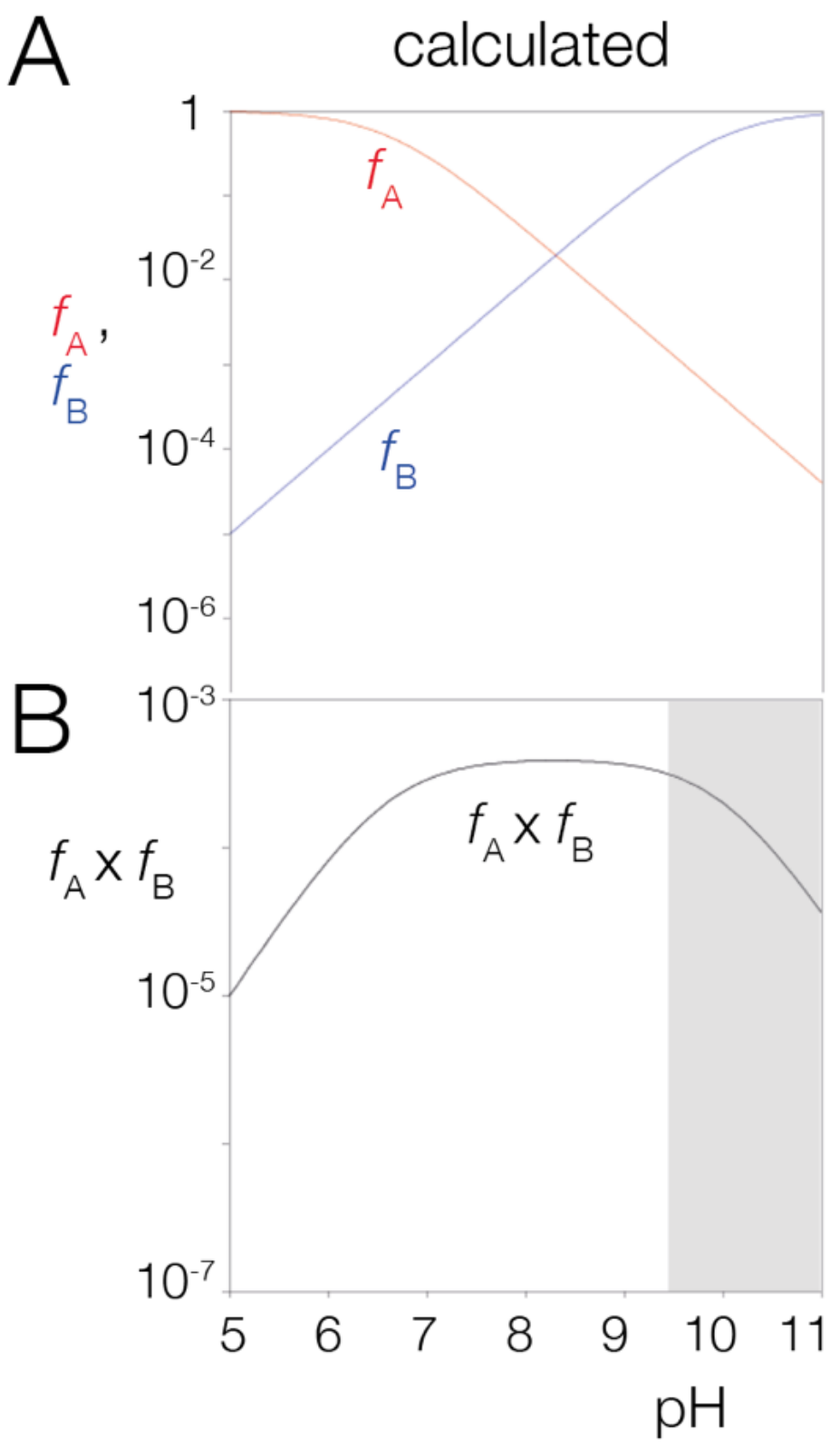

8. Fig 4A. Point 2: We are offered a mechanism in Figure 6E, in which OH_2 is the base deprotonating the 2'OH; indeed, on lines 239 and 245, it is implicated that if the general base is not a hydrated metal ion, it could be solvent water. However, this does not fit the rate-pH profile in Fig 4A at all. Indeed, if solvent water were the base, the rate pH profile would be inverted from that measured, with a low pH plateau and high pH decreasing arm. To get the observed rate-pH profile, the base needs to be OH- or it could be "due to deprotonation of the O2' itself" (line 341). However, both of these models have severe problems. The pK_a of OH- is 15.7 while that of O2' is similarly high. This means the $f_A x f_B$ will be 10^{-8} or so (the 10^{-4} from the above comment with another 10^{-4} from the difference in pK_a of OH- and $Mg(H_2O)$, which requires a k_{chem} of 10^7 s^{-1}, which is unrealistic when comparing to any RNase. This makes me favor general base catalysis by the metal ion, as originally proposed by Nakano. I would like the authors to consider that their metal ion could be both a Lewis acid and a general base, which Bevilacqua and Golden's data support, and try to model it as such. The authors will reply that if this is the case then the rate-pH profile should be like that in Fig S10 for TS. However, metal ion-RNA interactions are extraordinarily complex—much more so than in proteins—owing to the myriad interactions metal ions can have with the backbone. Perhaps Mg and Ca speed the reaction back up due to distal interactions, such as published at C41 in HDV by Bevilacqua and co-workers.

This is the most interesting question raised, and requires a response at length. Perhaps we can take this in two parts :

First we feel that the evidence for inner-sphere binding of Mg^{2+} ion is strong. The **F**o-**F**c map shows a strong peak around 2 Å from the non-bridging oxygen atom of the scissile phosphate (point 6 above), consistent with direct binding of the ion. For the *C. briggsae* ribozyme the activity requires a concentration of Co(III) hexammine considerably higher than the 2 mM Mg^{2+} used here (point 1 above) indicating that the Mg^{2+} ion is very tightly bound. PCB previously observed a stereospecific thio effect for the viral HDV ribozyme. Direct coordination of the Mg^{2+} ion to the phosphate oxygen atoms would be most consistent with a role as a Lewis acid catalyst, making the phosphorus a better electrophile.

That then leaves the question of what takes the proton from the O2' nucleophile ? At the present time this is difficult to answer with certainty and we need to be cautious in drawing any firm conclusions. f_A x f_B is easy to interpret when the general acid and base are components of the ribozyme; it is simply the fraction of active catalyst. But if a component of the solvent plays a role it is not so simple. PCB argues that this is likely to be hydrated metal ion. We would not exclude this possibility, but then we need to account for the lack of a dependence of cleavage rate on metal ion pK_a. We agree that the metal is in a complex electronegative environment in the RNA, yet the result stands in marked contrast to TS and pistol, where there is a very clear dependence of rate vs pK_a with a gradient close to 1 (see our Supplementary Figure S12). We also cannot exclude titration of another macromolecular component contributing to our observed pH dependence. We agree that water acting as the base is not consistent with our observed pH dependence. So at this time we prefer to leave this question essentially open. We have revised the Discussion to reflect these points.

9. Line 256. “C1” should be “N3”.

Corrected.

10. The 2 M Na^{+} experiments need large amounts of EDTA to assure that no contaminating divalents are driving the reaction, as first established in the HDV ribozyme by Been and co-workers.

The best grade of NaCl was used, with ~ 100 ppm contaminating ions. So it any other ion would not exceed 0.2 mM. However no difference in cleavage was observed ± 10 mM EDTA.

11. Line 372. When the authors write “although the O2’-P distance was good, at 3 Å” do they mean “was well determined, at 3 Å”?

What we mean is that the distance is good in terms of in-line fitness as defined by Breaker et al. To be maximal, the O2’-P distance should be 3 Å. It is very close to this value (hence “good” in this context). We have amended the text to “we found that its position deviated significantly from that required for an in-line attack, although the O2’-P distance was optimal, at 3 Å” which we hope clarifies this.

12. Line 399. 4.7 and 5.3 Å seem too far to be outer-sphere ligands to the metal ion. What precedent is there for such long distances?

Given the resolution of this structure these distances have an error associated. These distances will depend on the local geometry of the inner-sphere water molecule. But examination of metal ions bound to e.g. riboswitches (we have determined eight of these) 4.7 Å is within the range of metal-O or N distances, though at the upper end. Probably 5.3 Å is a little long, but probably within the likely error.

Minor Comments

1. Line 50. Delete the comma.

This has been done.

2. Line 68. I think "DNA species" should be "RNA species".

This has been corrected.

3. The title on the SI doesn't match that on the main text.

This has been corrected.

4. Line 143. The k_{obs} values should be added to Fig2 and/or legend.

The rate constants now been written above the progress curves for the cleavage reactions as requested.

5. Line 146. The 3 bp extension should be captured in the schematic.

The schematics shown in Figure 2 are not intended to show exact numbers of base pairs, but the P4 length is shown to be longer where the loop is open. If the reader needs exact lengths he/she can refer to Supplementary Table S1 presenting the complete sequences. We have added the sentence " In these schematics the base pairing bars do not represent the exact number of base pairs " to the legend for Figure 2 to make this clear.

6. Line 146. "The 5'end was extended by seven nucleotides from the expected cleavage site, so that the total length of the 5'RNA was 56 nt." There is no way to see this other than trust the authors. The nucleotide numbering should be shown in Figure 2.

The complete RNA sequences generated for the investigation of ribozyme activity are presented in Supplementary Table S1, including the seven nucleotide extensions.

7. Why did the authors switch from 5'-32P to 5'-fluorophore between J1/2 open and P4 open and what if anything is the consequence?

For a variety of reasons the lab switched from using ^{32}P to fluorophores at that time. It makes no real difference in terms of quantification.

8. Line 168. C57 is not in the major groove of P3, as it isn't part of P3. One might say it is NEAR the major groove of P3.

We would normally state that something (e.g. an α helix) can be inserted into the major groove of a helix. However, the P3 helix presents a rather flat surface so perhaps the preposition "in" is not the best here. We have therefore revised this to "the strand is juxtaposed with the major groove of P3". This is better, we agree.

9. Line 663. Remove the slash.

This is a matter of convention. We follow the format recommended by the RSC which is to write quantities in the form value / unit. In this case the unit is reciprocal time.

10. Line 411 delete ", and"

This is correct as written. It lists three kinds of evidence and the "and" brings in the third. So A, B and C.

Answers to editorial and reviewer points have been appended below written in blue

Dear Professor Lilley,

Thank you for submitting your manuscript "Lewis acid catalysis by a metal ion in a nucleolytic ribozyme; structure and mechanism of new HDV ribozymes" to Nature Communications. I am delighted to say that we are happy, in principle, to publish it under an open access license.

First, we ask you to revise your paper to address our editorial requests (in the attached Author Checklist) and any remaining comments from reviewers (included at the end of this email, if applicable).

Failure to comply with our editorial requests will result in further revisions and delays in accepting your manuscript. Please also see the *Nature Communications* formatting instructions for further information.

FEATURED IMAGE

If you wish, you can provide an interesting image (but not an illustration or schematic) for consideration as a Featured Image on the Nature Communications homepage. The file should be 1200x675 pixels in RGB format and should be uploaded as a Related Manuscript File with the title “featured image suggestion”. In addition to our home page, we may also use this image (with credit) in other journal-specific promotional material. If your featured image is chosen you will need to complete a Licence to Publish form which will be sent to you via DocuSign at a later stage.

We have generated a Featured Image suggestion, and uploaded it. It shows the active site of the *C. briggsae* HDV ribozyme prior to cleavage. The legend for this is : The active center of the *C. briggsae* HDV ribozyme prior to cleavage is shown. C57 acts as a general acid to protonate the leaving group while a bound magnesium ion acts as a Lewis acid to activate nucleophilic attack.

OPEN ACCESS

Nature Communications is a fully open access journal. Articles are made freely accessible on publication. For further information about article processing charges, open access funding, and advice and support from Nature Research, please visit http://www.nature.com/ncomms/about/open-access

At acceptance, you will be provided with instructions for completing the open access license agreement on behalf of all authors. This grants us the necessary permissions to publish your

paper. Additionally, you will be asked to declare that all required third party permissions have been obtained, and to provide billing information in order to pay the article-processing charge (APC).

ORCID

Nature Communications is committed to improving transparency in authorship. As part of our efforts in this direction, we are now requesting that all authors identified as 'corresponding author' create and link their Open Researcher and Contributor Identifier (ORCID) with their account on the Manuscript Tracking System (MTS) prior to acceptance. ORCID helps the scientific community achieve unambiguous attribution of all scholarly contributions. For more information please visit http://www.springernature.com/orcid
For all corresponding authors listed on the manuscript, please follow the instructions in the link below to link your ORCID to your account on our MTS before submitting the final version of the manuscript. If you do not yet have an ORCID you will be able to create one in minutes.
https://www.springernature.com/gp/researchers/orcid/orcid-for-nature-research
Huang (0000-0002-2121-365X) and Lilley (0000-0001-6882-2818) both have ORCID IDs. Link on web site ?

IMPORTANT: All authors identified as 'corresponding author' on the manuscript must follow these instructions. Non-corresponding authors do not have to link their ORCIDs but are encouraged to do so. Please note that it will not be possible to add/modify ORCIDs at proof. Thus, if they wish to have their ORCID added to the paper they must also follow the above procedure prior to acceptance.
To support ORCID's aims, we only allow a single ORCID identifier to be attached to one account. If you have any issues attaching an ORCID identifier to your MTS account, please contact the Platform Support Helpdesk.

PRESENTATION OF AUTHOR NAMES USING NON-ROMAN CHARACTERS

Nature Portfolio journals can support presentation of author names using non-Roman characters in the online version of the article. If you wish to, please include author names in parentheses after the Roman-character spelling; https://www.nature.com/articles/s41550-021-01509-7>see example online here. Currently supported scripts are: Arabic, Chinese, Cyrillic, Devanagari, Greek, Hebrew, Hangul, Japanese and Persian. Should the manuscript be accepted, you will be asked to verify the rendering is correct at proof stage.

POLICIES

In recognition of the time and expertise our reviewers provide to Nature Communications's editorial process, we formally acknowledge their contribution to the external peer review of articles published in the journal. All peer-reviewed content will carry an anonymous statement of peer reviewer acknowledgement, and for those reviewers who give their

consent, we will publish their names alongside the published article. For more information, please refer to our FAQ page at https://www.nature.com/documents/ncomms-reviewer-information.pdf

Nature Portfolio journals encourage authors to share their step-by-step experimental protocols on a protocol sharing platform of their choice. Where such protocols are available, please provide a DOI or other citation details in the paper. Nature Portfolio's *Protocol Exchange* is a free-to-use and open resource for protocols; protocols deposited in *Protocol Exchange* are citable and can be linked from the published article. More details can found at https://www.nature.com/protocolexchange/about

AUTHOR CHANGES DURING PREVIOUS REVISIONS
If there have been any changes to the author list since your initial submission, please use this approval form www.nature.com/documents/nr-author-list-change-form.pdf, arranging for all authors on your paper to sign the statement confirming that they agree to the author list being changed, and add this document to your resubmission. Please also add an explanation of the changes to your cover letter.

There has been none

SUBMISSION
Please use the following link to submit the required items:
https://mts-ncomms.nature.com/cgi-bin/main.plex?el=A6S4FGWU2B7cDM7I5A9ftdpcxjwoZy5d8SPHBedqqQZ
** If you wish to forward this email to your coauthors, please delete the link above **

If you are planning an embargoed press release, please let the handling editor know in your cover letter.

We look forward to seeing your revised manuscript. Please let us know if the process may take longer than two weeks.

Best regards,

Minju Ha, PhD
Senior Editor
Nature Communications

REVIEWERS' COMMENTS

Reviewer #1 (Remarks to the Author):

The authors have addressed all my points and I support publication without reservation.

Thank you.

Reviewer #3 (Remarks to the Author):

Comments on other Reviewers
Reviewer 1
1.) Response to Reviewer #1, Point 6. A double bond is needed between N3 and C4. As drawn, C4 doesn't have an octet. This is particularly important as this is now the concluding figure of the paper.

We agree. We have re-drawn the chemical structure in Figure 6 to include a partial double bond on C57.

Reviewer 2
1.) The dialogue on Line 171 is quite interesting. I encourage the authors to write a future perspective on ribozymes concerning this feature.

Thanks for the suggestion. We have recently reviewed the structures of riboswitches and discussed multiple base interactions in some detail [L. Huang and D. M. J. Lilley Some general principles of riboswitch structure and interactions with small-molecule ligands *Quart. Rev. Biophys*. **e13**, 1–22 (2025)].

My Review
1.) I appreciate the authors conducting the new experiment to test for inhibition by cobalt hexamine. Given that they (effectively) find no inhibition (Ki>10 mM), then I agree that their mechanism, with direct coordination of the 2'O to the Mg2+ ion, is realistic. At the same time, it is important that they acknowledge that the outcome of this inhibition was different in the original HDV ribozyme (Ki of 45 uM in Science 2000, footnote 22), which suggests that the mechanistic role for the metal ion might differ between HDV ribozymes. I think this should be mentioned.

We have added this caveat to the text

2.)3.) I agree with changes.

Thanks

4.) I think this explanation is fine, but it is important to point out that these considerations are at neutral pH. We can always take the pH down to 4 and make chemistry the rls, as Harris, Piccirilli, and York have successfully done.

That's interesting, thanks

5.) The new additions strengthen the paper and are really appreciated. Thank you.

Thanks

6.) The new Fo-Fc maps significantly strengthen the paper, and the authors' honest grappling with the limitations of the data are appreciated.

Thanks. We very much appreciated the suggestion to include a better image of the **F**o-**F**c map for the ions. We feel this greatly strengthens our conclusions.

7.) This now looks good. Glad this was caught!

I made a silly mistake uploading the wrong file. Thank you for catching this !

8.) I'm satisfied that the authors write "We agree that water acting as the base is not consistent with our observed pH dependence". And of course, it is not necessary to know everything to publish (or none of us would ever publish). I'm satisfied with this change. With that said, it is also possible that the proton transfer agent is buffer/metabolite, as we demonstrated under certain conditions for twister (Messina and Bevilacqua, 2018) and others have protein enzymes. The authors may want to entertain this possibility and indeed test it in a future study.

Thanks. We will indeed think about this for future studies.

9.)-12.) I agree with changes.

Thanks

Minor Revisions: I agree with changes.

Thanks

Review signed: Phil Bevilacqua
Date: July 14, 2026

We are grateful to all the reviewers, but especially Phil Bevilacqua who raised a lot of important and interesting points. As a result of this we believe the paper is greatly improved. This is the peer review process working at it very best !
