# Supplementary material for: Structure and mechanism of HDV ribozymes revealing Lewis acid catalysis by a metal ion
Source: Nat Commun. 2026 Sep 2;17:10462. doi: 10.1038/s41467-026-77452-w (PMC13635239; doi:10.1038/s41467-026-77452-w)
Supplement: Supplementary file 4 [file 41467_2026_77452_MOESM4_ESM.zip › Source data file -2.docx]

Gel files

All gels used for presentation as figures, or for quantification of rates presented in this paper are supplied here. These are the images directly from the phosphorimager/fluorimager as .tif files. The have not been cropped. Their intensity has been uniformly increased (in Photoshop using “curves”) simply to make bands visible here; otherwise they are too feint to see. In some gels there is a very fast-migrating band present in all tracks (including zero time) that arises from the fluorophore used, It is not related to ribozyme action. In some gels marker dyes are visible on the left. All data were acquired in the laboratory by David Lilley, and the files stored in his computer.

**1. Gel electrophoretic images that appear in the Figures of the paper**

**Figure 2**

**Fig 2** (top right) *C. briggsae* L1/.2 open reaction progress in Mg^2+^ ions (phosphorimaging ^32^P)

HDV EXPT / IMAGES / HDVcb WT+mut 1224 / HDVCb timco W +2mut 1224-[Phosphor].tif

**Fig 2** (bottom left) *Ackermannviridae* P4 open reaction progress in Mg^2+^ ions

HDV EXP / IMAGES / 201225 / 201225 B AkHDVrz -[Alexa Fluor 532].tif

Two sets of data were electrophoresed on this gel; the left side only was used for the Figure.

**Fig 2** (bottom right) *C. briggsae* P4 open reaction progress in Mg^2+^ ions

HDV EXP / IMAGES / 061025 / 61025 HDV Mg C-[FAM].tif

**Figure 5**

**Fig. 5A** *C. briggsae* and C57U mutant

HDV EXP / IMAGES / 23625 / HDVCballmut_23725c-[FAM].tif

The left side of this gel contains additional (non-relevant) tracks not shown in the Figure.

**Fig. 5B** *C. briggsae* multiple divalent cations

HDV EXP / IMAGES / 051025 / HDV 51025 5 ions B-[FAM].tif

**Fig. 5C** *C. briggsae* G25A and G25O^6^S

HDV EXP / IMAGES / 20126 / 4126 B HDVCb muts-[Alexa Fluor 488].tif

**2. Replicates for measurement of kinetic** **rates**

*C. briggsae* P4 open reaction progress in Mg^2+^ ions (4 replicates, two gelsx2 time courses).

Reaction times : 4x 0, 2, 4, 8, 20, 40 s

HDV EXP / IMAGES / 020102526 / 251002 / HDV Mg gel1 B-[FAM].tif and 251002 HDV Mg gel 2 B-[FAM].tif

*C. briggsae* P4 open reaction progress in Ca^2+^ ions (4 replicates, two gelsx2 time courses).

Reaction times: 4x 0, 2, 5, 10, 35, 60 s

HDV NEW / HDV EXP / IMAGES / 260925 / 250926 HDV Ca C-[FAM].tif and 250926 HDV Ca.tif

*C. briggsae* P4 open reaction progress in Mn^2+^ ions (4 replicates, two gelsx2 time courses)

Reaction times: 4x 0, 2, 5, 10, 35, 60 s

HDV NEW / HDV EXP / IMAGES / 270925 / 250927 HDV Mn B-[FAM].tif and 250930 HDV Mn B-[FAM].tif

*C. briggsae* P4 open reaction progress in Mg^2+^ ions in MES (pH 6.0 left, and 6.7 right)

Reaction times: 2x 0, 1, 3, 10, 20, 40, 60 s

HDV NEW / HDV EXP / IMAGES / 130925 / 250913 HDV 2pH-[FAM].tif

*C. briggsae* P4 open reaction progress in Mg^2+^ ions in MES (pH 6.0) One gelx2 time courses)

Reaction times: 2x 0, 3, 10, 20, 40, 60 s

HDV NEW / HDV EXP / IMAGES / 160925 / 250916 HDV 2xpH6.0 C-[FAM].tif

*C. briggsae* P4 open reaction progress in Mg^2+^ ions in MES (pH 6.7) One gelx2 time courses)

Reaction times: 2x 0, 3, 10, 20, 40, 60 s

HDV NEW / HDV EXP / IMAGES / 170925 / 250917B HDV 2xpH6.7-[FAM].tif

*C. briggsae* P4 open reaction progress in Mg^2+^ ions in Tris (pH 8.5). (4 replicates, two gelsx2 time courses)

Reaction times: 4x 0, 2, 4, 8, 15, 40 s

HDV NEW / HDV EXP / IMAGES / 291125 / 291125 pH8.5 B CbHDV-[FAM].tif and 31225 B HDV pH 8.5 3,4 -[FAM].tif

**3. Supplementary Figures**

**Fig. S1.** Site-specific cleavage of the *C.briggsae* and *Ackermannviridae* ribozymes, with marker RNA for the products.

Left. This gel contains both *C.briggsae* (fluorescein) and *Ackermannviridae* (Cy3) samples, but was fluorescein was excited at 488 nm. HDV NEW / HDV EXP / IMAGES / 30726 / 307 HDV Cb Ac prod 488-[Alexa Fluor 488].tif

Right. This contains only *Ackermannviridae* (Cy3) samples, excited at 532 nm HDV NEW / HDV EXP / IMAGES / 1826 / 18 HDV Ack prod balan-[Alexa Fluor 532].tif

**Fig. S9.** Effect of a C58U mutation on the catalytic activity of the *Ackermannviridae* HDV ribozyme

HDV NEW / HDV EXP / IMAGES / 291225 / 291225 B AkHDV C58-[Alexa Fluor 532].tif

The last three lanes were shown in the figure S8, and inverted left-to-right.

**Fig. S10**. Reaction progress of the *C.briggsae* HDV ribozyme in 2 mM Mn^2+^ or Ca^2+^ ions at 37°C.

Mn^2+^ data

HDV NEW / HDV EXP / IMAGES / 270925 / 250927 HDV Mn B-[FAM].tif

The six lanes on the left of the gel were shown in Figure S9.

Ca^2+^ data

HDV NEW / HDV EXP / IMAGES / 300925 / 250930 HDV Ca B-[FAM].tif

The six lanes on the left of the gel were shown in Figure S9.

**Fig. S11**. Reaction progress of the *C.briggsae* HDV ribozyme in 2 M Na^+^ ions.

HDV NEW / HDV EXP / IMAGES / 171025 / 171025E 6.7 HDV Na-[FAM].tif

**Fig. S12**. Reaction progress of the *C. briggsae* HDV ribozyme G25 O^6^S.

HDV NEW / HDV EXP / IMAGES / 090925 / 250909_HDV 6SG-[FAM].tif

The left six lanes of this gel were shown in Figure S11.
